# Leveraging machine learning to consolidate the diversity in experimental results of perovskite solar cells†

**DOI:** 10.1039/d3ra02305b

**Published:** 2023-07-25

**Authors:** Wahid Hussain, Samina Sawar, Muhammad Sultan

**Affiliations:** a Department of Physics, Quaid-i-Azam University 45320 Islamabad Pakistan wahidhussaiin@gmail.com; b Department of Plant Sciences, Quaid-i-Azam University 45320 Islamabad Pakistan; c Department of Physics, Kohsar University Murree 47150 Punjab Pakistan muhammad.sultan@kum.edu.pk

## Abstract

Perovskite solar cells offer great potential for smart energy applications due to their flexibility and solution processability. However, the use of solution-based techniques has resulted in significant variations in device fabrication, leading to inconsistent results on the same composition. Machine learning (ML) and data science offer a potential solution to these challenges by enabling the automated design of perovskite solar cells. In this study, we leveraged machine learning tools to predict the band gap of hybrid organic–inorganic perovskites (HOIPs) and the power conversion efficiency of their solar cell devices. By analyzing 42 000 experimental datasets, we developed ML models for perovskite device design through a two-step predicting method, enabling the automation of perovskite materials development and device optimization. Additionally, band gap dependence of device parameters from experimental data is also validated, as predicted by the Shockley–Queisser model. This work has the potential to streamline the development of perovskite solar cells (PSCs) and optimize their performance without relying on time-consuming trial-and-error approaches.

## Abstract 2

Application of a machine learning approach to device design. Starting from database analysis followed by a dataset creation based on those insights. Data preprocessing is done to extract features for ML prediction and design new PSCs.

## Graphical abstract

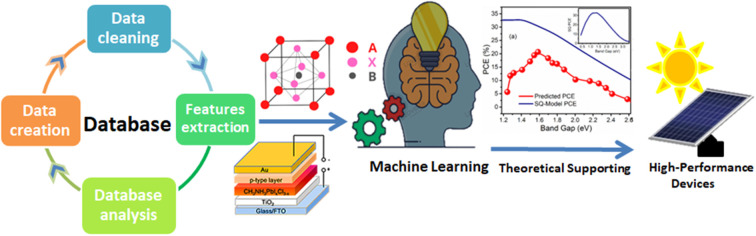

## 1 Introduction

Fossil based energy sources are declining along with their destructive environmental impacts. Solar cells are considered one of most sustainable sources of clean energy. The solar cells market is dominated by silicon (Si), which has reached an efficiency of 26% for single crystal solar cells and 22% for poly-crystal solar cells.^1^ Although the price of Si has declined substantially in recent years, this technology still has a minor share in the overall energy market. Purification chemistry, high-temperature processing, and advanced manufacturing techniques, present significant challenges for Si solar cells.^2^

Secondly, contemporary smart applications require solar cell to be flexible, having absorber layer capable of harvesting selective part of light spectrum. Therefore, scientific community is investigating for next generation solar cells such as organic, dye-sensitized, and PSCs. The PSCs have demonstrated the rapid growth in power conversion efficiency (PCE), increasing from 3.2% in 2009 to 25.2%^3^ in 2021 over a 12 year period.

A variety of materials have been investigated for various types of layers in PSCs, including electrodes, electron-transport layer (ETL), hole-transport layer (HTL), and perovskites. For fabrication of PSCs one-step deposition, two-step deposition, and other synthesis procedures have also been developed and reported earlier.^4^ There is no other solar cell technology that enables excellent device performance while still being affordable, efficient, and flexible. Stability is still the main hindrance to the commercialization of these solar cells. In addition, other issues have also been examined such as device hysteresis, toxicity, and reproducibility.^5^ Significant technical and basic research efforts are being undertaken to address these issues. Numerous experimental studies have reported the mitigation of consequences caused by degradation. However, there remains a significant gap in prediction methods that can effectively guide the synthesis process. Furthermore, researchers have generated extensive amounts of experimental and simulation data to address various device problems, thereby laying the foundation for utilization of data-driven approaches in development and optimization.

With the advent of artificial intelligence (AI) and machine learning (branch of AI), remarkable progress has been made in materials science since the Materials Genome Initiative (MGI) by USA.^6^ To better understand the physics underlying the properties of materials and find ways to enhance them, materials scientists are constantly striving to develop tools and approaches. The trial-and-error techniques for material synthesis and characterization are time and money-consuming.^7^ Machine learning has been extensively used in all the domains of materials science such as superconductors,^8^ magnetic materials,^9^ crystal system prediction and design,^10^ and last but not the least, the materials for solar cells such as organic solar cells^11^ and perovskite solar cells.^12^

Recent advancements in perovskite solar cells have led to the introduction of ML in PSCs. Simultaneously, there has been a notable increase in publications on the use of machine learning for perovskites,^13^ with expanding applications across different avenues.^14^ In one study Andreas *et al.*^15^ used fingerprinting descriptors to estimate the efficiency of organic solar cells and discovered a mean absolute error of 0.07. Also Yuta *et al.*^16^ predict the PCE of organic solar cells using physical and fingerprinting descriptor. Fingerprinting descriptors, also known as molecular descriptors, are numerical representations of chemical compounds or molecules. For perovskite solar cells performance, Cagla *et al.*^17^ performed machine learning using various descriptors (HTL, ETL, additives, annealing time, and temperature, *etc.*) to see the trends in the research data by performing statistical analysis. In a recent study Jinxin *et al.*^18^ used composition of materials, band gap, and the energy difference between VBM and CBM were employed to perform predictive analysis for device efficiency and band gap. However, due to the complexity of how device performance depends on several physical properties and other factors, it is difficult for machine learning to predict the efficiency of devices.

To achieve efficient application of ML in the development and autonomous prediction of device performance, without relying on experimental and computational methodologies, substantial work is essential in terms of descriptors, quality, and quantity of data. Although the machine learning models developed by Jinxin aimed to predict PCE based on the energy differences of layers such as VBM and CBM levels, they did not include the corresponding layers such as HTL and ETL. As different HTL and ETL have been used with the same energy differences in various devices and led to different results. Therefore, it is important to include those layers in the descriptors to establish a meaningful relationship between the device structure and its PCE. For the first time, we use this technique of integrating device layers to develop ML models for perovskite solar cells. This communication aims to bridge the gap between device design and automation by using the rational strategy in choosing the descriptors. According to FIAR data principle^19^ the descriptor must be useful, applicable to all situations, reproducible, and easily accessible.

## 2 Methods

In this work we analyzed the perovskite database and exhibit the key insights. We prepare a sub-data by using the extracted knowledge from the database and leverage machine learning techniques to develop various models such as Linear Regression (LR), Ridge regression (RR) k-nearest neighbors (KNN), random forest (RF), and XGBoost. An autonomous two-step predicting method for selection of perovskites and other device layers is proposed for optimization and development of new PSCs by predicting the band gap, PCE, and other device parameters including FF, *V*_oc_, and *J*_sc_.

These models utilize descriptors such as absorber material composition, band gap, device stack layers (electron transport layer, hole transport layer, substrate layer, back-contact), device architecture (nip, pin), and device area to predict and design PSCs. Unlike other ML approaches for predicting and designing PSCs, these models eliminate the need for prior calculations or experimentation for descriptors values. Instead, one simply needs to select the desired stoichiometry ratio of perovskite composition for perovskite absorber layer to achieve a suitable band gap. Additionally, models for device parameters prediction will assist in determining the appropriate device layers (HTL, ETL), for optimal device design.

In the Perovskite Database Project, Jesper and Eva^20^ collated a database of about 42 000 data samples containing perovskite devices that have been explored and studied in the literature. However, the whole data in the database is very scattered and therefore could not be able to utilize in data-driven approaches. Therefore data cleaning and data wrangling were needed to perform in order to make complex data into a refined form so that it can be utilized in ML approaches.

**Fig. 1:**
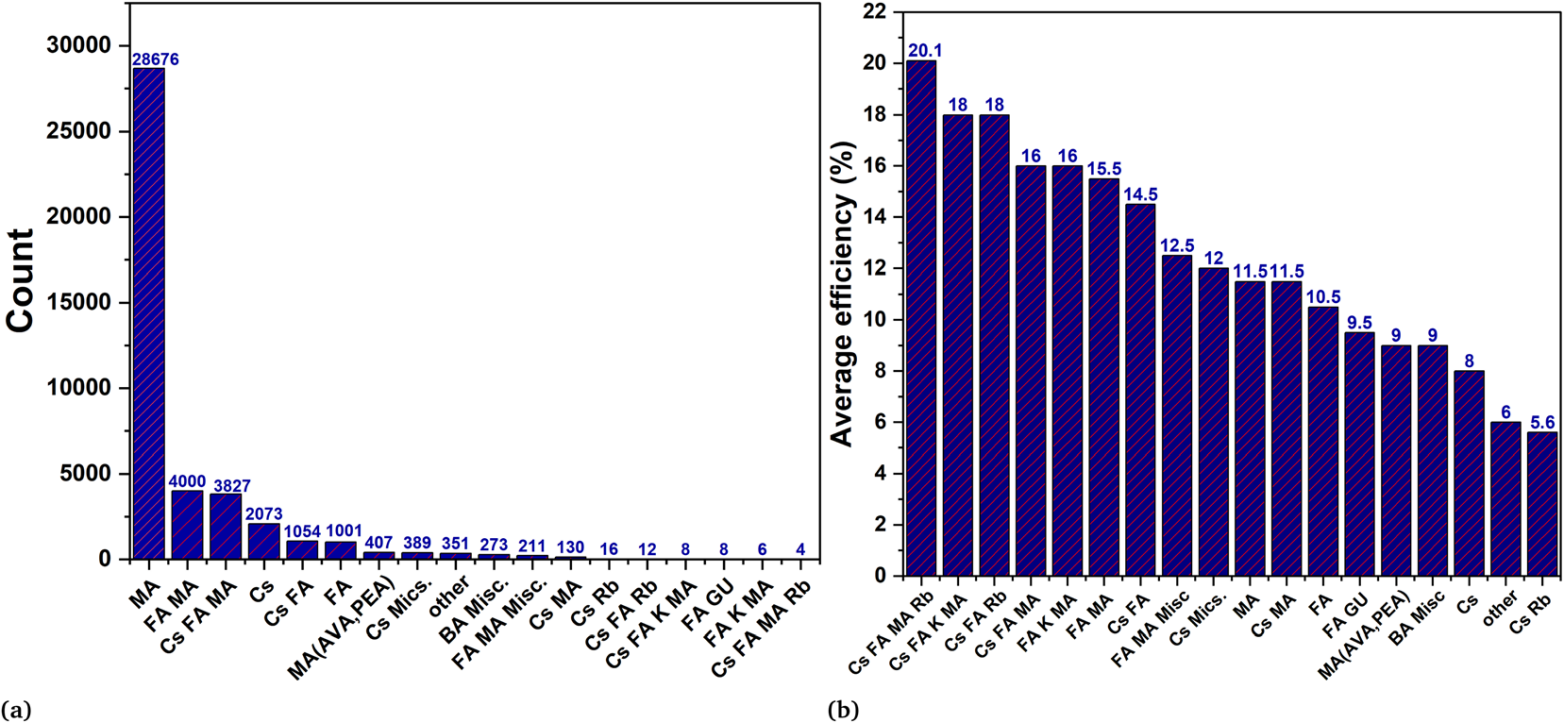
Perovskite database analysis. In figure (a) the count of respective perovskite is mentioned while figure (b) represent the average accuracy of different perovskite in descending order.

Fig. 1(a) indicates the device data of different perovskites with their count. The dataset includes devices with varying stoichiometry compositions of mixed cations and anions at each site (A, B, X) of the ABX_3_ perovskite structure. Most of the device data have duplicated samples with different outcomes such as MAPbI_3_ has almost 29 000 device data. It has gained significant attention in research due to being one of the first perovskite materials studied for its potential application as a photovoltaic absorber. However, there are merely a few numbers of reports using MAPbI_3_ with an efficiency of more than 20%. Despite extensive work on MAPbI_3_, it has fatal shortcomings such as low stability and toxicity due to moisture sensitivity with volatile nature, and lead (Pb) respectively.^21,22^

Similarly, other most widely used single cation perovskites as shown in the count plot such as FA, Cs also exhibit phase stability issues at room temperature.^23,24^ In order to overcome these problems, the mixing of cations and halides has been an essential topic for researchers. Combinations of various monovalent cations at A-site (MA, FA, Cs, Rb, K) with divalent metal cations at B-site (Pb, Sn, Bi, Sb) and anions at X-site (Br, I, Cl) of ABX_3_ perovskite structure have been analyzed. These analyses are done by considering the sizes of the cations and anions with tolerance factor (*t*) which is empirically use to estimate the perovskite crystal structure.^25^1
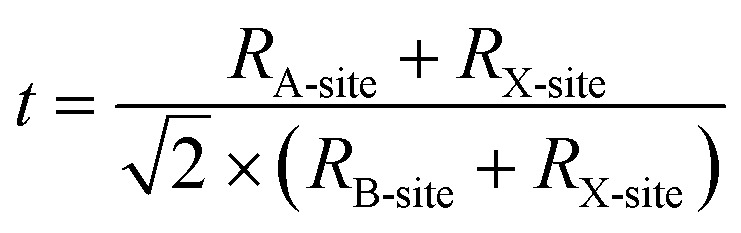
where *R*_A-site_, *R*_B-site_, *R*_X-site_ indicates the radius of A-site, B-site cations and X-site anions respectively. According to *t* value the black phase bulk perovskite structure forms when the tolerance factor is between 0.8 and 1.^26^

Fig. 1(b) highlights the importance of mixing cation and halides on the PCE of the device. It is evident that quadruple cation and triple cation are outperforming others followed by double cation in terms of overall performance and efficiency. Cs, MA, and FA based perovskites are only examples of single-cation perovskites that exhibit a black phase and high PCE. However, it should be noted that their pristine structures are inherently unstable. Also, Rb being near to the optimal *t* value, can be incorporated into the feasible perovskite structure by employing the mixing of different cations.^27,28^ In contrast to the pure form of MA and FA perovskites, the mixing strategy of the double cation (MA, FA) elucidates that adding a modest amount of MA to the mixture, results in the best crystallization with an induced photoactive black phase.^29^ In the same way mixing of cations (Cs, Fa) induced photo and moisture stability along with photovoltaic performance.^30,31^ The inclusion of small cations such as Cs or Rb suppresses the halide segregation and causes the wide band gap perovskite for tandem solar application.^32^

The triple cations perovskites (MAFACsPb(I/Br)_3_) as indicated in the count Fig. 1(a) have been conveniently studied among all the potential combinations of various materials at various sites. These triple cation perovskites were created in order to enhance crystal quality, stability, reproducibility (mainly ignored), and efficiency.^33^ These investigations also pave the way for the inclusion of Rb, K, and Na as a fourth cation, leading to the formation of quadruple cation perovskites. According to Fig. 1(b), the quadruple cations (Cs, K, FA, MA) device recorded the highest average PCE.

## 3 Data preparation

**Fig. 2:**
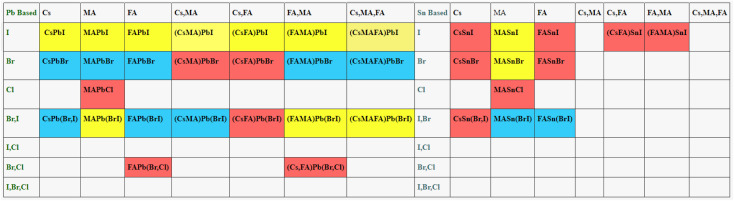
The matrix of various simple and mixed cations and mixed halides produced by the permutation of A = (MA, FA, Cs) and X = (I, Br, Cl). The matrix consists of two types of B-site cation Pb and Sn. The perovskite materials highlighted in yellow represent the mixing of both Pb and Sn and have also been studied extensively. The materials composition in blue is also investigated widely. The red ones are also investigated to some extent and white boxes are never explored or least investigated.

As the machine learning approaches mainly depend on the quality and quantity of data therefore data should be free from sparsity and duplicates in order to develop generalizable machine learning models. The selected perovskites for the dataset are shown in the matrix Fig. 2. The yellow and blue boxes indicate extensively studied Pb and Sn based perovskites particularly triple cation perovskite due to their suitability of optoelectronic properties. Also, within yellow boxes, double and triple cation perovskites contain mixed ratio of Pb and Sn. The red ones are comparatively less investigated. The blank or white boxes show that these materials were never studied or least studied.

The data also contains duplicated samples, therefore need to be removed and only one point can be considered otherwise duplicates samples tend to bias the machine learning models.^34^ The data in the database for perovskite characteristics and features inherently lack homogeneity. With this approach, it is impossible to gain insights into issues such as why perovskites with a single or a group of similar material qualities have varied electrical or optical properties.^35^

Constructing a model using this data would present a formidable challenge. Because inclusion of all perovskite absorber materials and device layer materials, even after eliminating duplicate samples, would result in an immense feature space. Such a vast number of features could potentially lead to the curse of dimensionality,^36^ further complicating the modeling process.

A thorough investigation and analysis is done to select the data with distinct and mostly investigated perovskite materials along with the most frequent device layers (electron transport layer, hole transport layer, backcontact layer, substrate layer). We use one-hot encoding method for the descriptors of all the device layers. This encoding method assigns a value of 1 to indicate the presence of a layer and 0 to indicate its absence. For the perovskite absorber layer we use compositional (A = Cs, FA, MA; B = Pb, Sn; X = Br, I, Cl) descriptors which represent the stoichiometry ratio of these materials.

## 4 Data insights

**Fig. 3:**
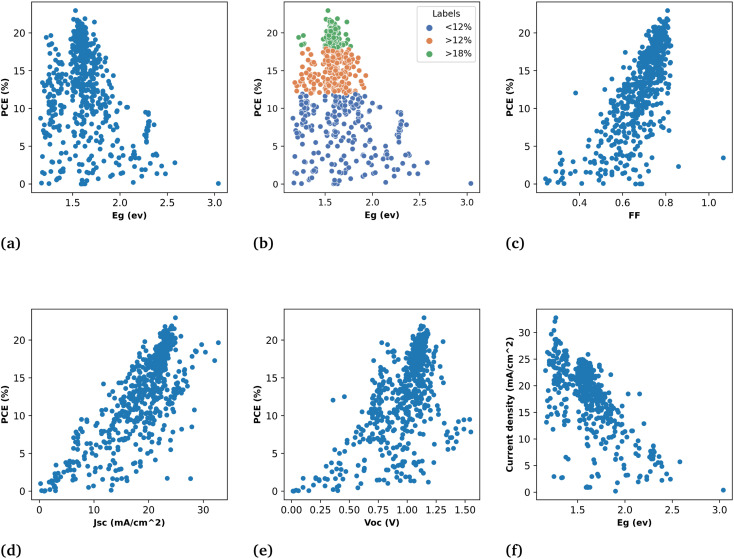
Schematics of refined data statistics used in this work for modeling band gap and PCE. (a) PCE plot against band gap, (b) PCE plot against band gap with efficiency label, (c) PCE plot against FF, (d) PCE plot against *J*_sc_, (e) PCE plot against *V*_oc_, and (f) current density *versus* band gap.

In Fig. 3 the statistics of device parameters (613 samples) is represented after the post processing of the original data (42 300 samples) and summarized the insights of our data. These insights confirm that the extracted data aligns with both experimental and theoretical trends, exhibiting no outliers. This ensures the data quality and makes it suitable for the development of models.

Fig. 3(a) is plotted against PCE and band gap of the perovskite materials. As it is shown that the most high-efficiency devices belong to the band gap values near to or equal to 1.51 eV which is the optimal value of band gap for perovskites. In Fig. 3(b) we exhibit the plot of PCE against band gap with efficiency labels where green dots represent the devices with efficiency >18% while blue dot represents devices with efficiency <12% and so on.

The power conversion efficiency of a solar cell device is obtained from the following expression.2
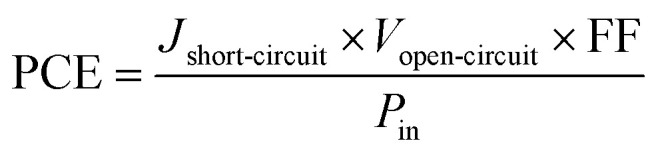
where *J*_short-circuit_ (*J*_sc_) is short circuit current density, *V*_open-circuit_ (*V*_oc_) is open circuit voltage, FF is fill factor, and *P*_in_ is the incident illumination power. According to eqn (2) PCE equally depends on *J*_sc_, *V*_oc_, FF. However, *J*_sc_ in Fig. 3(d) have more influence on the PCE. It shows a linear dependence on PCE and also the trade-off relationship between *J*_sc_ and *V*_oc_ have been established through band gap. Although the decrease in band gap increases the current density but lowers the *V*_oc_ of the device which is the manifestation of the tradeoff relationship. There are some devices that have high PCE with high *J*_sc_ value but low *V*_oc_. Hence, the high value of *V*_oc_ is essential but not the only condition for high PCE. For most efficient devices *V*_oc_ lies between 1 and 1.3 in Fig. 3(e).

The FF in Fig. 3(c) shows a linear relationship with PCE from 0.4–0.81 but the colinearity between PCE and *J*_sc_ is more prominent compared to FF. The cell width has also demonstrated that fill factor and short-circuit current density are engage in a trade-off with each other. If the cell width is too small it will have less *J*_sc_ due to the small area on the other hand if the cell width is too large the resistance in the cell will diminish the FF. Therefore, the cell area plays a pivotal role in PCE.

In order to see the relationship between device parameters and perovskite properties, a *J*_sc_ plot is drawn against the band gap of perovskites in Fig. 3(f). As can be seen from the trend of the plot the increase in current density with the decrease in band gap is consistent with the expectations. The narrow band gap materials can absorb the longer wavelength of the infrared region of the solar spectrum and more energy can be harvested from the sun. Before applying ML approaches, it is crucial to analyze the data. We remove all the incorrect data points and outliers to develop the effective models.

## 5 Results and discussion

### 5.1 Band gap prediction

**Fig. 4:**
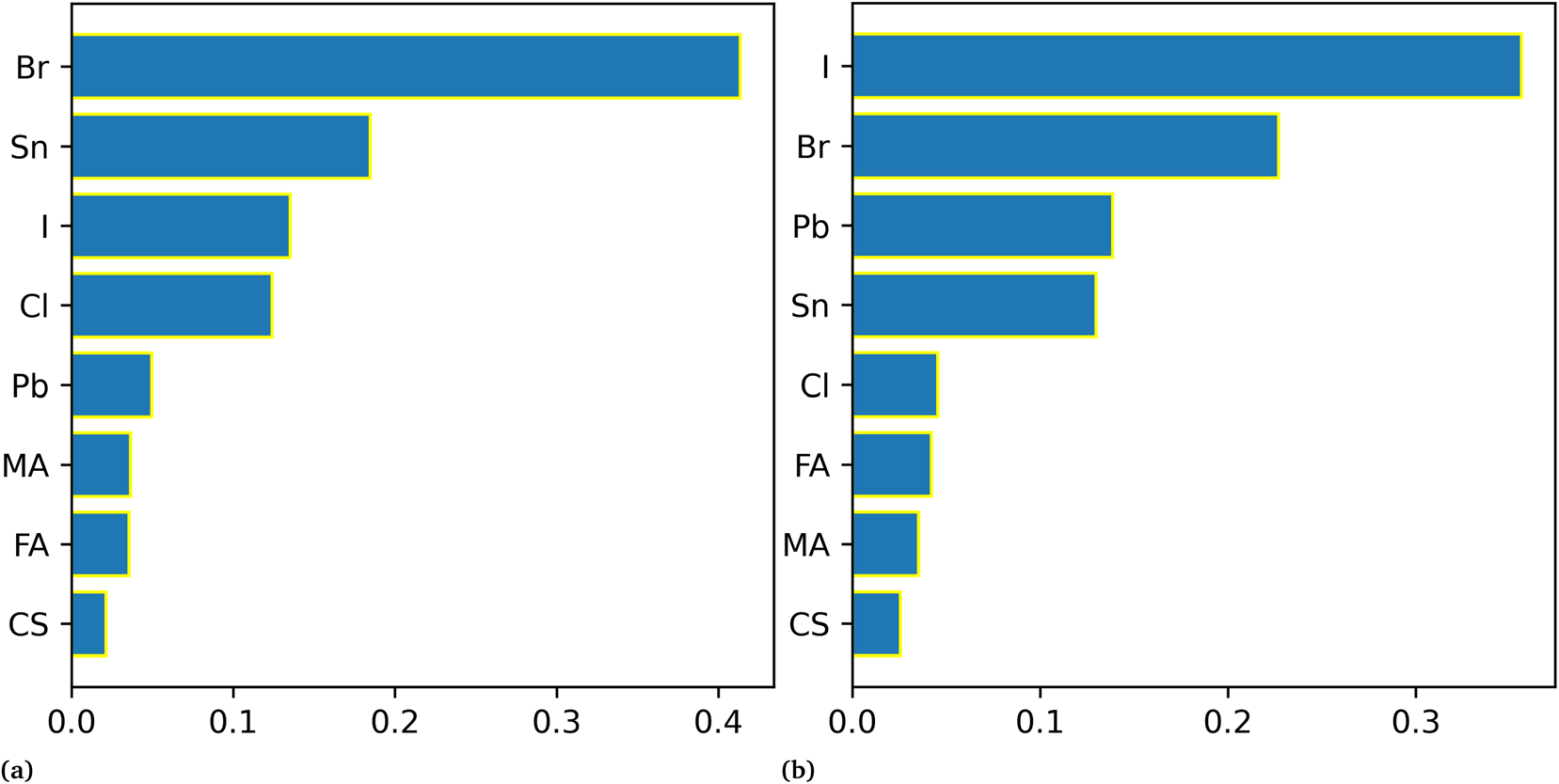
Impact of different materials on band gap prediction. (a) is for the RR model and (b) is for RF.

For the band gap prediction, we choose Cs, MA, and FA as A-site cations, Sn, and Pb as B-site cations, and Br, Cl, and I as X-site anions of the ABX_3_ structure of the perovskite. The different stoichiometry mixing compositions of these materials at each site is taken as input variables for the different machine learning models and band gap as the output of the corresponding perovskite. It spans almost all the possible stoichiometry concentrations of the single, double, and triple cations perovskite. This will allow us to design the optimal perovskite absorber layer with desired band gap by selecting the required stoichiometry composition. Our most ML model results resonate with the theoretically established consensus on tuning the band gap by either mixing halogen or divalent B-site metallic cations. As shown in Fig. 4(a) and (b) that the models gave the least importance to organic–inorganic cations and exhibit more dependence on halogens and B-site metallic cations for band gap prediction.

### 5.2 Device parameters prediction

**Fig. 5:**
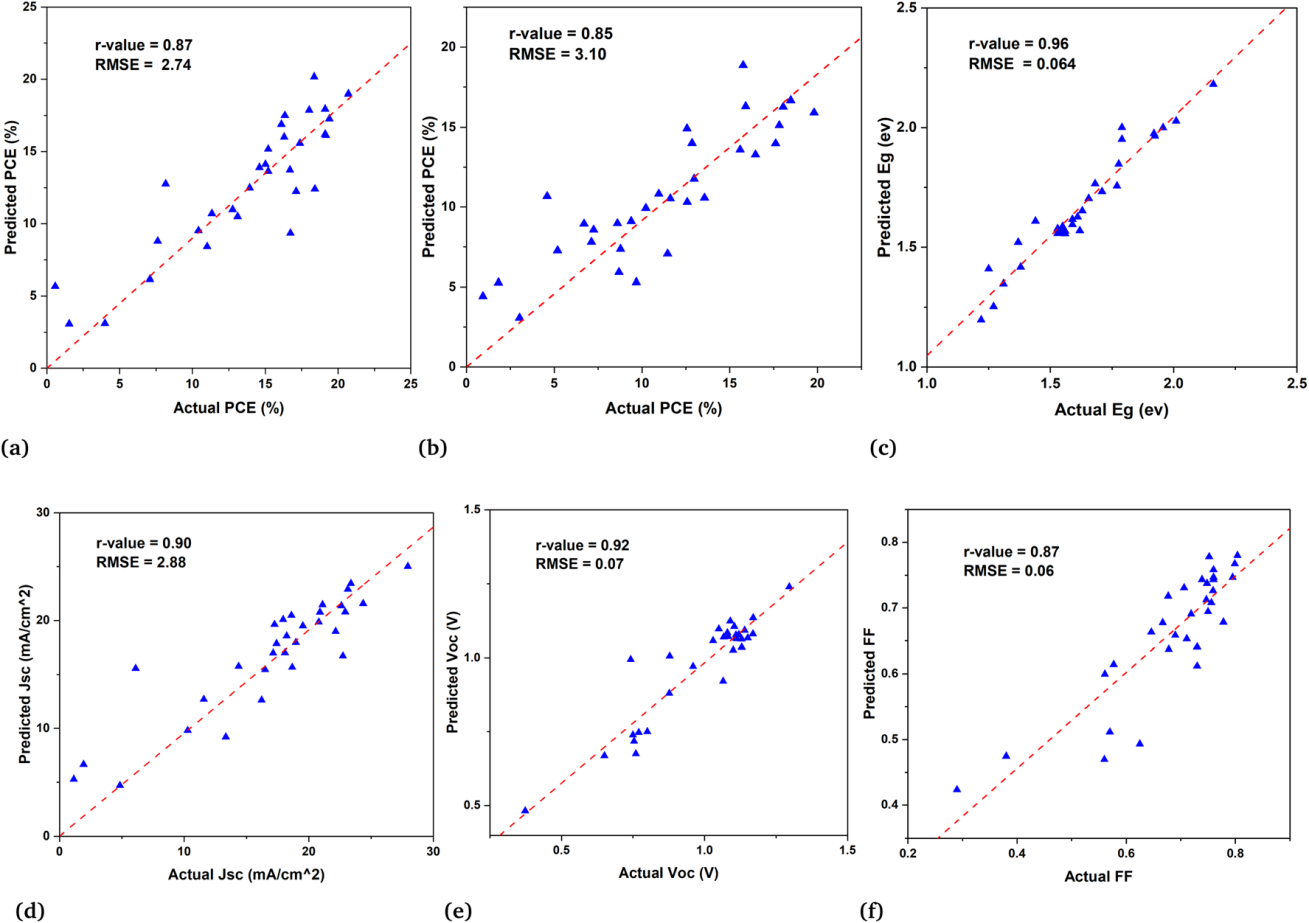
Demonstration of results of different ML models used in this work for predicting band gap and PCE. Figure (a) is the direct prediction of PCE, (b) shows the calculation of PCE from predicted values of *J*_sc_, *V*_oc_, and FF, and the prediction of the band gap is shown in figure (c). Also, the device parameter prediction are shown; (d) actual *J*_sc_*versus* predicted *J*_sc_ (e) actual *V*_oc_*versus* predicted *V*_oc_, and (f) actual FF *versus* predicted FF.

In the second subset of data the models are built to predict the PCE of PSCs along with predictions of other parameters such as current density (*J*_sc_), fill factor (FF), and open circuit voltage (*V*_oc_). These models for device parameter prediction will help us in screening the optimal HTL and ETL. As eqn (2) shows that PCE is obtained from these parameters. Therefore, we predict the PCE directly using ML models and also calculated the PCE from the ML predicted values of *J*_sc_, FF, and *V*_oc_ in order to see the ML model's ability to generalize for various outcomes with the same input descriptors. This finding suggests that the curated data and extracted descriptors we used for ML are more accurate in representing the underlying patterns. The results of both approaches are given in Table 1. It shows that the machine learning result for both approaches is quite similar for RF and XGBoost models. Fig. 5 is a demonstration of the fact that our models were able to learn the patterns in the data and indicate their reliability to predict PCE instantly and efficiently.

**Table 1:** Table comprises the ML model type with their corresponding result for band gap prediction, PCE direct prediction from the input data, and PCE calculated from predicted values of *J*_sc_, *V*_oc_ and FF

Models	Band gap prediction		PCE direct prediction		PCE calculated from predicted *J*_sc_, *V*_oc_, FF values	
	*r*-Value	RMSE (eV)	*r*-Value	RMSE (%)	*r*-Value	RMSE (%)
LR	0.80	0.155	0.77	3.5	0.58	5.18
RR	0.93	0.087	0.82	3.5	0.76	3.38
KNN	0.91	0.099	0.84	3.43	0.67	4.09
RF	0.94	0.068	0.86	2.92	0.83	3.35
XGBoost	0.96	0.064	0.88	2.41	0.85	3.01

Among 692 data samples, there are duplicates and sparse samples which we filter out to ensure the quality of data. Even though removing the duplicates causes the data to shrink to 613 samples but still the results of machine learning models have proven to be very efficient for predicting high-efficiency devices. The direct PCE prediction from the input data is shown in Fig. 5(a) and demonstrates a remarkably well match between actual PCE and predicted PCE. Despite the fact as mentioned above and also in Fig. 3(b) very few devices included in the data has PCE > 18% still it predict well with high precision almost all the devices having efficiency >18%.

The Pearson correlation matrix between input variables is shown in Fig. 1 of ESI.† The matrix gives the linear correlation between variables. More positive value mean strong positive correlation while more negative value means strong negative correlation. Also for evaluation of random forest model the SHAP value plot is given in Fig. 2 of ESI.† The plot quantifies how different features affect the model output in prediction. Figure shows that band gap is the most important feature for the PCE prediction which is well established in experiment and theory and shows the power of machine learning approach.

### 5.3 Autonomous layers design strategies

The autonomous perovskite solar cell device design is proposed and adopted by employing two-step prediction method. In the first step band gap prediction will help in the design of perovskite absorber layer of new PSCs. The ML model for band gap prediction will give the suitable stoichiometry composition of different materials for perovskites absorber layer.

In the second step, the prediction of PCE, combined with the design of the perovskite absorber layer in the first step, aids in the screening of ETLs and HTLs. This approach eliminates the need for prior experimental or simulation values for the descriptors of these models. As a result, this method automates the design of these layers and eliminates the need for time-consuming and resource-intensive methods.

The tunable band gap is one of the important characteristics of the perovskites which can be done either by mixing of B-site metallic cations or halogens. This property makes perovskites suitable for a plethora of applications such as tandem solar cells,^37–39^ light emitting devices,^40^ laser,^41^ photodetector,^42^ and X-ray or particle detection.^43^ Because optoelectronic properties primarily depends on the band gap of the absorber material and PSCs require an ideal and suitable perovskite compound as an absorber. Therefore, band gap prediction is essential in the first step.

**Fig. 6:**
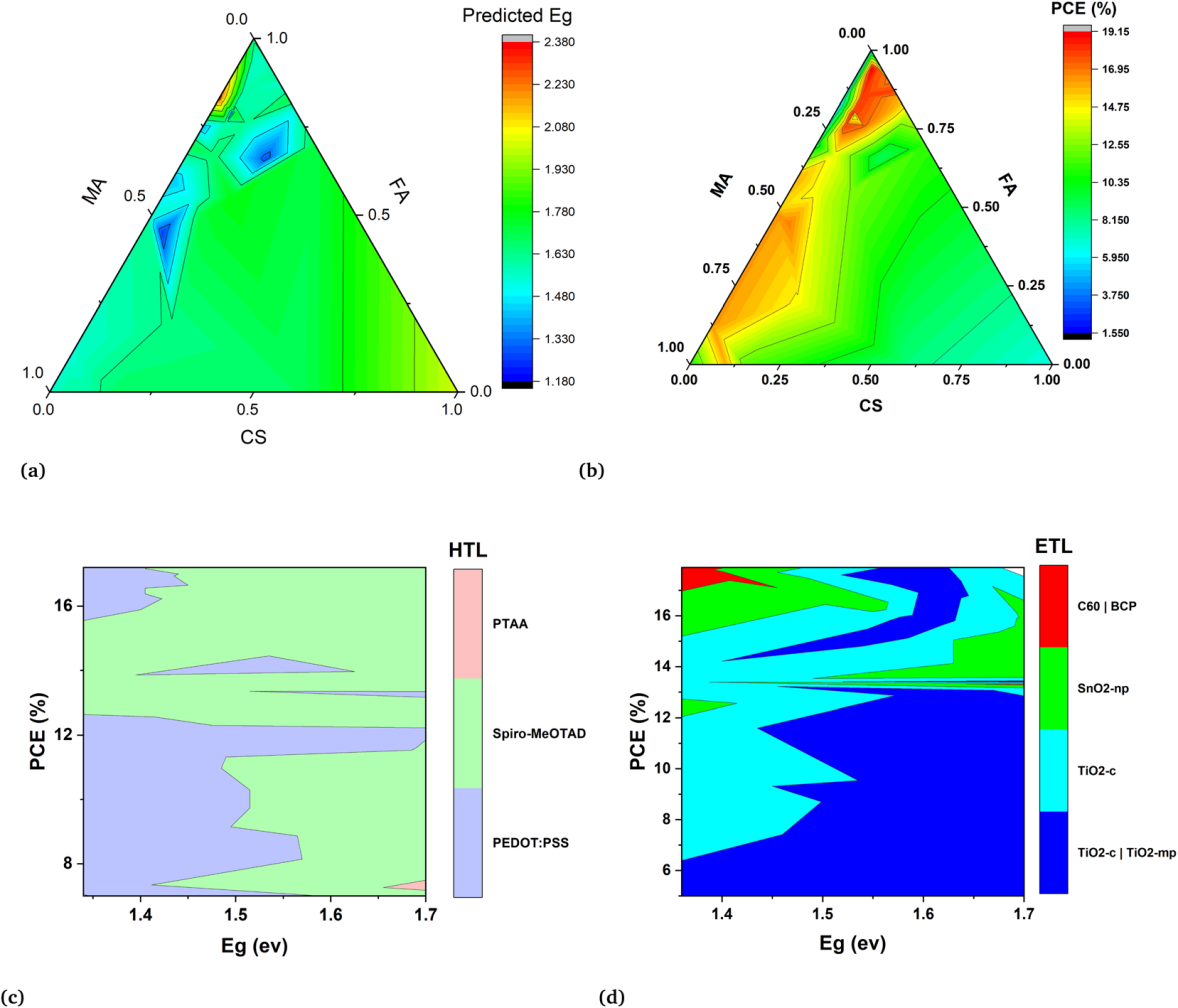
The 2d contours are obtained for optimal range of band gap for high efficiency devices. (a) The illustration of band gap prediction of mixed cation perovskites with the different mixing ratio of A-site (A = Cs, FA, MA), B-site (B = Pb, Sn), and halogen (X = Br, I, Cl). (b) Demonstrates the PCE prediction of mixed cation perovskites with a different mixing ratio of A-site (A = Cs, FA, MA), B-site (B = Pb, Sn), and halogen (X = Br, I, Cl). (c) Shows best predicted HTL and their relation with optimal range values of band gap for high PCE devices. Similarly, in (d) electron transport layers were obtained.

The results of the band gap prediction, as depicted in Fig. 6(a), indicate that perovskites falling within the red region of the contour are chloride-based or bromide-based perovskites. These findings align well with established experimental results. The highest band gap value is given by the chloride-based perovskite such as CsPbCl_3_ has *E*_g_ = 3.04. Similarly, as we increase the bromine content in the perovskite compound the band gap will increase accordingly.^44^ Another important point is captured by the ML prediction of the band gap values that the Sn-based perovskites have low band gap values which are shown in the blue area of the contour. As we know from previous studies band gap tuning can also be possible by mixing Pb and Sn with increased content of Sn leads to a low band gap.^45^ Although, A-site cations do not directly affect the band gap nature, understanding their shape, radius, and charge distribution is crucial for optimizing the overall performance of the perovskite layer in the device. This trend is also evident in machine learning predicted results.

In Fig. 6(b) the high efficiency devices are represented by the red region of contour. It shows us that these solution base devices correspond to a high mixing ratio of FA cation and small mixing of cesium cation with a range of mixing ratios of MA cation. All the red region devices are obtained by keeping the Pb = 1 and iodine as halogen with no or small mixing of bromine which resonates with the experimental studies so far. They indicate that for a more reliable device the more suitable perovskite compound should have less content of bromine and MA due to blue penalty and stability issues respectively. The low PCE of the devices in the blue region of the contour are single cations devices based on single cations either Cs, MA, or FA. Most of them have no or less iodine content which means they are mostly bromide-based single cation devices.

In the second step ETLs, and HTLs are screened out by predicting the PCE and other parameters of devices such as *J*_sc_, *V*_oc_, and FF. The hole transport and electron transport layer play a pivotal role in optimizing the perovskite devices by reducing the surface recombination at each interface of the absorber layer. The three hole transport layers such as PTAA, PEDOT:PSS and spiro-MeOTAD are screened for the models development and these layers are also widely studied in perovskites devices. The contour in Fig. 6(c) is obtained for the optimal range (1.3–1.7 eV) of the band gap suggested in the theory for optoelectronic properties. It can be seen that PEDOT:PSS is best for low band gap perovskites while spiro-MeOTAD is optimal for band gap values >1.46 eV. The electron transport layers (ETLs) are also evaluated and their relationship with the absorber layer through the band gap is determined to optimize perovskite devices. The selection of ETL depends on the desired band gap of the perovskite material. As we know, the most optimized band gap for perovskites is 1.51 eV and TiO_2_-c—TiO_2_-mp is the ETL that has been extensively used for these devices and further confirmed by the model prediction.

### 5.4 Comparison of ML prediction with theory

**Fig. 7:**
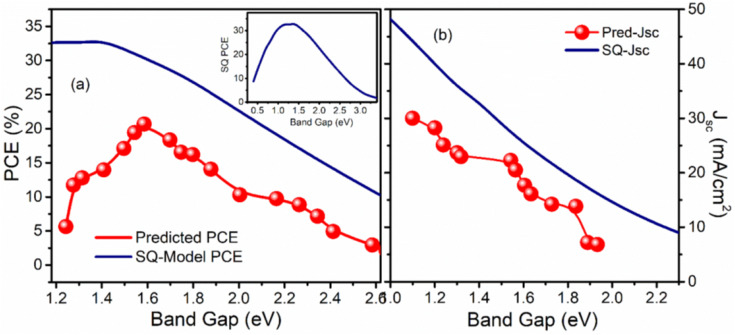
Illustration of results of XGBoost model used in this work for predicting band gap and PCE and their comparison with theory. Panel (a) and (b) exhibits the comparisons of the predicted PCE and current density respectively with the limit proposed by Shockley and Queisser in their theory, brown and yellow lines represent the maximum PCE. The insets show the reduced size curves of the S–Q theoretical limit.

The maximum PCE of a single junction solar cell is limited by the Shockley–Queisser (S–Q) limit. A curve comparing band gap and PCE is generated in Fig. 7(a) to validate the machine learning model against the theoretical curve proposed by Shockley and Queisser.^46^ The ML model successfully captured the trend proposed by Shockley and Queisser in their theory.^47^ However, the maximum PCE and its corresponding band gap in the model do not precisely align with the theory due to practical solar cell limitations caused by non-radiative losses. Shockley and Queisser's theory is reliant on a number of assumptions, one of which is that the only type of recombination that occurs in solar cells is radiative recombination, which depicts the ideal condition of the solar cell. In real-case scenarios, solar cells have many non-radiative recombination mechanisms which causes less practical maximum PCE contrary to the proposed maximum PCE in theory.

Auger recombination, Shockley–Read–Hall (SRH) recombination, and surface recombination^48^ are the three major non-radiative recombinations. Auger recombination^49^ is exist even in pure semiconductors mainly in indirect semiconductors where radiative recombination of electrons and holes requires larger momentum which cannot be provided by the emitted photon. Alternatively, the momentum along with energy is given to the other electron in the conduction band which leads it to an even more high energy state. This mechanism gains significance with an increased charge carrier density, which is less common in perovskites under illumination. However, it can restrict the performance of PSCs when exposed to concentrated light. The SRH recombination takes place due to the trap states caused by defects.^50^ There are various origins of these defects such as imperfect crystal, impurities in the crystal, grain boundaries, *etc.*

It should also be noted that the highest efficiency devices in the dataset have a band gap from 1.5 eV to 1.6 eV which might indicate the fact that perovskites devices are optimized in this range of band gap and are not fully optimized in the low band gap range suggested by the theory. This might be the reason to give the peak value at 1.51 eV by the machine learning model instead of the theoretical value of 1.3 eV.

Further, the comparison of predicted short circuit current density with the theoretical values is also displaced by the curve in Fig. 7(b) which quite astonishingly matches the trend as well as the maximum of the current density curve despite including all the non-radiative recombination. From the machine learning model results, we can argue that the recombination mechanism has little effect on current density as compared to *V*_oc_ which then causes the reduction of the PCE. From the S–Q theory, we know that when we increase the band gap current density decreases assuming that there is no non-radiative recombination.

## 6 Conclusion

We analyzed the perovskite database in order to see the trend in data for high-efficiency perovskite solar cell devices. We concluded that the most efficient and stable devices are based on triple and quadruple cations perovskites. One of the major concerns of reproducibility from the commercialization point of view is seemed to be resolved by these devices. In particular, triple-cation HOIPs are structurally less complex compared to quadruple-cations perovskite solar cells. We established a dataset based on these devices and analyzed to validate its insights for the data quality and integrity. We screened out the unique 696 materials among 42 300 for perovskite band gap prediction and 613 for the PCE prediction.

An autonomous two-step predicting method is proposed, aiming to streamline the design process of new PSCs. This method eliminates the need for extensive experimental and simulation work, effectively reducing the time-consuming and resource-intensive aspects of PSCs design. Our work for the first time paves the way to automate the process of design and fabrication. This work is indeed endorsing the power of machine learning for the development and optimization of high-efficiency devices, otherwise it is practically impossible to analyze such vast information except through trial and error which causes high cost and slow development.

## Author contributions

WH – conceptualization, methodology, validation, investigation, data curation, formal analysis, software, visualization, writing–original draft, writing – review & editing; SS – formal analysis, software, visualization; MS – conceptualization, resources, visualization, supervision.

## Conflicts of interest

The authors declare no competing interests.

## Supplementary Material

RA-013-D3RA02305B-s001
