# Supplementary material for: Leveraging machine learning to consolidate the diversity in experimental results of perovskite solar cells
Source: RSC Adv. 2023 Jul 25;13(32):22529–37. doi: 10.1039/d3ra02305b (PMC10367956; doi:10.1039/d3ra02305b)
Supplement: RA-013-D3RA02305B-s001 [file RA-013-D3RA02305B-s001.pdf]

Electronic Supplementary Material (ESI) for RSC Advances.
This journal is © The Royal Society of Chemistry 2023

Supporting information

Leveraging Machine Learning to Consolidate the Diversity in Experimental Results of Perovskite Solar Cells

Wahid Hussain[a], Samina Sawar[b], Muhammad Sultan[c,*]

[a] *Department of Physics, Quaid-i-Azam University, 45320, Islamabad, Pakistan*

[b] *Department of Plant sciences, Quaid-i-Azam University, 45320, Islamabad, Pakistan*

[c] *Department of Physics, Kohsar University Murree, 47150, Punjab, Pakistan*

*Corresponding author: muhammad.sultan@kum.edu.pk

Figure 1: Figure 1 shows the correlation matrix of input variables. The correlation value range is from 1 to -1. Where positive value means there is a positive linear correlation between the two variables while negative values indicate the negative correlation between them.

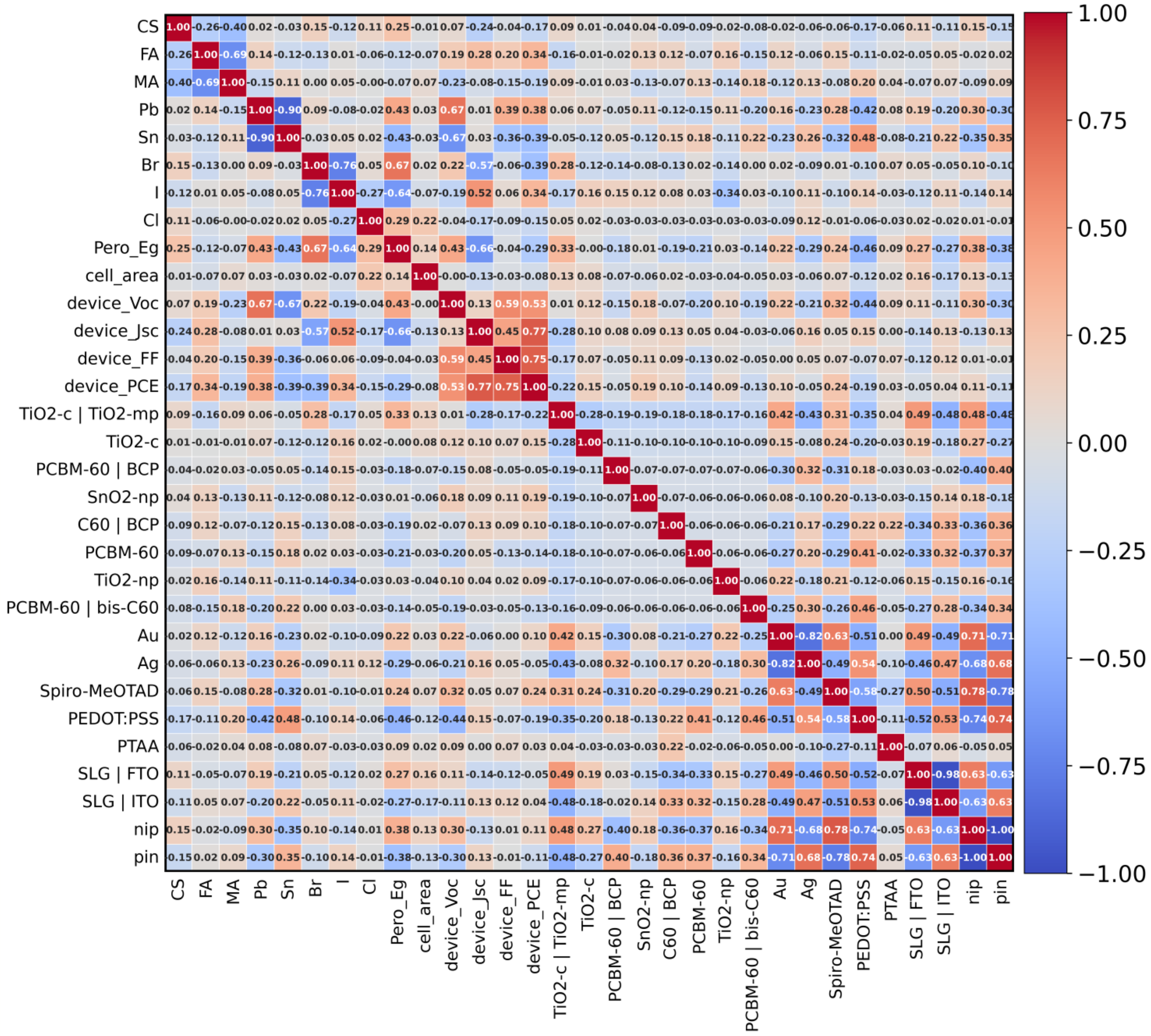

Figure 2: Figure 2 illustrate the SHAP value of XGBRegressor model output for PCE prediction. The color bar represents the features importance value in descending order. The density and each color point represent the number of samples. Also the color bar indicates the feature importance in descending order.

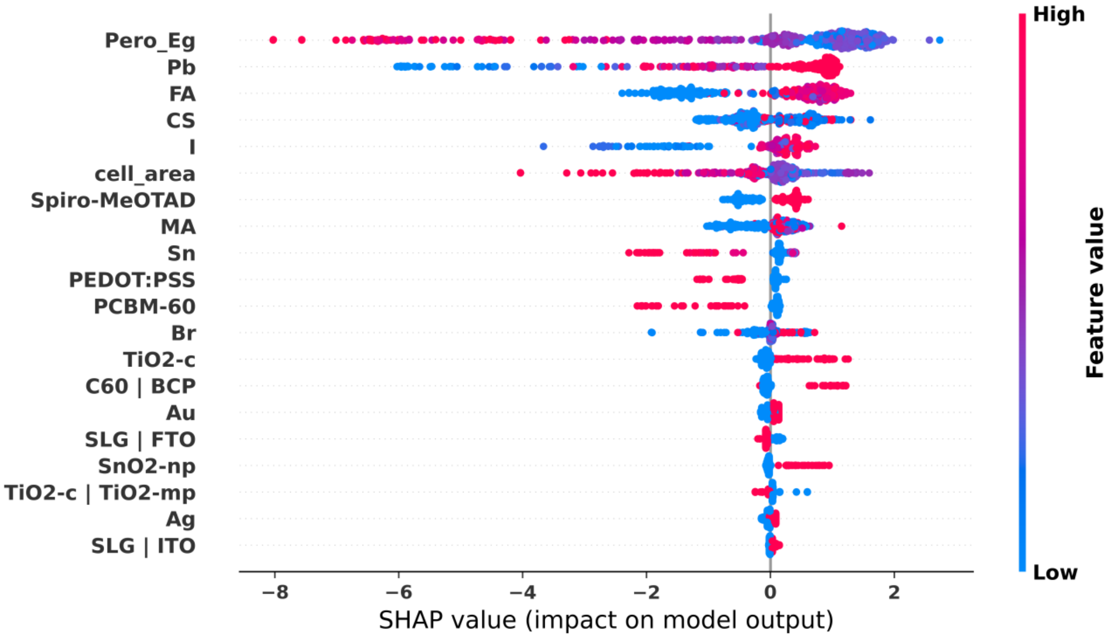

Perovskite database:

The full data having 42000 data samples can be obtained by using the following link.
Perovskite database

Curated refined band gap dataset:

Dataset obtained from perovskite database for band gap prediction it contains 1106 data samples . Also the PCE dataset would be made available upon request.

CS	FA	MA	BA	Pb	Sn	Sb	Bi	Br	I	Cl	Pero_Eg
0	0	0	2	1	0	0	0	0	1	0	2.35
0	0	0.75	0.5	1	0	0	0	0	1	0	1.88
0	0	0.67	0.67	1	0	0	0	0	1	0	1.81
1	0	0	0	0	1	0	0	0	1	0	1.27
1	0	0	0	0	1	0	0	1	0	0	1.75
1	0	0	0	0	1	0	0	1	2	0	1.37
1	0	0	0	0	1	0	0	2	1	0	1.65
1	0	0	0	0	1	0	0	0.5	2.5	0	1.375

1	0	0	0	0	1	0	0	0.6	2.4	0	1.4
1	0	0	0	0	1	0	0	2.4	0.5	0	2.36
1	0	0	0	1	0	0	0	0	1	0	1.73
1	0	0	0	1	0	0	0	1	2	0	1.92
1	0	0	0	1	0	0	0	1	0	0	2.3
1	0	0	0	1	0	0	0	2	1	0	2.07
1	0	0	0	1	0	0	0	0.15	2.85	0	1.7
0.003	0	0	0	0.997	0	0	0	1	0	0	2.2
0.005	0	0	0	0.995	0	0	0	1	0	0	2.29
0.01	0	0	0	0.99	0	0	0	1	0	0	2.11
0.03	0	0	0	0.97	0	0	0	1	0	0	1.89
1	0	0	0	1	1	1	1	2	1	0	2.08
1	0	0	0	1	0	0	0	0.09	2.91	0	1.77
1	0	0	0	1	0	0	0	0.21	2.79	0	1.81
1	0	0	0	0.96	0	0	0.04	0	1	0	1.45
1	0	0	0	0.1	0	0	0.9	0	1	0	1.22
1	0	0	0	0.3	0	0	0.7	0	1	0	1.29
1	0	0	0	0	0	0	1	0	1	0	1.78
1	0	0	0	1	0	0	0	1	1	0	1.77
1	0	0	0	0.9	0.1	0	0	2	1	0	1.79
1	0	0	0	1	0	0	0	1.5	1.5	0	1.7
1	0	0	0	1	0	0	0	2.4	0.1	0	2.38
1	0	0	0	1	0	0	0	2.4	0.2	0	2.35
1	0	0	0	1	0	0	0	2.4	0.3	0	2.32
1	0	0	0	0	0	0	1	1	0	0	2.2
1	0	0	0	0.98	0	0	0	0	1	0	1.71
1	0	0	0	0.95	0	0	0	0	1	0	1.75
1	0	0	0	0.93	0	0	0	0	1	0	1.8
1	0	0	0	0.9	0	0	0	0	1	0	1.84
1	0	0	0	0.85	0	0	0	0	1	0	1.93
1	0	0	0	1	0	0	0	0.31	2.69	0	1.75
1	0	0	0	0.5	0.5	0	0	1	2	0	
1	0	0	0	0.9	0	0	0	1	2	0	1.895
1	0	0	0	0.8	0	0	0	1	2	0	1.889
1	0	0	0	0.7	0	0	0	1	2	0	1.881
1	0	0	0	0	0.05	0	0	0	2.95	0	
1	0	0	0	0.94	0	0	0	1	2	0	
1	0	0	0	0.9	0	0	0	2	1	0	1.88
1	0	0	0	0.88	0	0	0	1	2	0	
1	0	0	0	1	0	0	0	0.75	2.25	0	1.79
1	0	0	0	1	0	0	0	1.9	1.1	0	1.7
1	0	0	0	0.98	0	0	0	1	2	0	1.919
1	0	0	0	0.97	0	0	0	1	2	0	1.9
1	0	0	0	1	0	0	0	1	0.12	1.88	1.85
1	0	0	0	1	0	0	0	1	0.22	1.78	1.85
1	0	0	0	1	0	0	0	1	0.28	1.72	1.85
1	0	0	0	1	0	0	0	1	1.88	0.12	1.85
1	0	0	0	1	0	0	0	1	1.78	0.22	1.85

1	0	0	0	1	0	0	0	1	1.72	0.28	1.85
1	0	0	0	0.97	0	0	0	1	0	0	2.27
1	0	0	0	0.03	0	0	0	1	0	0	2.27
1	0	0	0	1	0	0	0	0.45	2.55	0	1.84
1	0	0	0	1	0	0	0	0.2	2.8	0	1.82
1	0	0	0	0.6	0.4	0	0	0	1	0	1.38
0.05	0.95	0	0	1	0	0	0	0.15	2.85	0	1.48
0.1	0.9	0	0	1	0	0	0	0.3	2.7	0	1.48
0.15	0.85	0	0	1	0	0	0	0.45	2.55	0	1.48
0.2	0.8	0	0	1	0	0	0	0.6	2.4	0	1.48
0.25	0.75	0	0	1	0	0	0	0.6	2.4	0	1.68
0.25	0.75	0	0	1	0	0	0	0	1	0	1.58
0.17	0.83	0	0	1	0	0	0	0.8	2.2		1.72
0.17	0.83	0	0	1	0	0	0	1.2	1.8		1.79
0.17	0.83	0	0	1	0	0	0	1.5	1.5		1.86
0.17	0.83	0	0	1	0	0	0	1.8	1.2		1.93
0.2	0.8	0	0	1	0	0	0	0.9	2.1		1.75
0.17	0.83	0	0	1	0	0	0	1.2	2.8		1.75
0.13	0.87	0	0	1	0	0	0	1	2		1.72
1	0.8	0	0	1	0	0	0	0.42	2.58		1.59
0.2	0.8	0	0	1	0	0	0	1.2	1.8		1.77
0.17	0.83	0	0	1	0	0	0	0.17	0.83		1.6
0.15	0.85	0	0	1	0	0	0	0.12	2.88		1.6
0.17	0.83	0	0	1	0	0	0	0.6	2.4		1.61
0.3	0.7	0	0	1	0	0	0	1	0		2.287
0.1	0.9	0	0	1	0	0	0	1	0		2.277
0.2	0.8	0	0	1	0	0	0	0.75	2.25		1.67
0.05	0.95	0	0	1	0	0	0	0	1		1.48
0.1	0.9	0	0	1	0	0	0	0.3	2.7		1.62
0.4	0.6	0	0	1	0	0	0	1.05	1.95		1.8
0.25	0.75	0	0	1	0	0	0	0.3	2.7		1.57
0.125	0.875	0	0	1	0	0	0	0.375	2.625		1.6
0.15	0.85	0	0	1	0	0	0	0.81	2.19		1.72
0.25	0.75	0	0	1	0	0	0	0.6	2.4		1.72
0.17	0.83	0	0	1	0	0	0	0.51	2.49		1.64
0.25	0.75	0	0	1	0	0	0	0.51	2.49		1.63
0.25	0.75	0	0	0.5	0.5	0	0	0	1		1.24
0.15	0.85	0	0	1	0	0	0	2.1	0.9		2
0.2	0.8	0	0	0.75	0.25	0	0		1		1.35
0.2	0.8	0	0	0.5	0.5	0	0		1		1.3
0.2	0.8	0	0	0.25	0.75	0	0		1		1.2
0.2	0.8	0	0	0	1	0	0		1		1.2
0.15	0.85	0	0	0.375	0.625	0	0		1		1.24
0.25	0.75	0	0	0.37	0.6255	0	0		1		1.24
0.2	0.8	0	0	0.3	0.7	0	0		1		1.35
0.3	0.7	0	0	0.7	0.3	0	0		1		1.3
0.25	0.75	0	0	0.5	0.5	0	0		1		1.27
0.4	0.6	0	0	1	0	0	0	0.9	2.1		1.76

0.1	0.9	0	0	1	0	0	0		1		1.509
0.2	0.8	0	0	1	0	0	0		1		1.515
0.3	0.7	0	0	1	0	0	0		1		1.523
0.5	0.5	0	0	1	0	0	0	0.51	2.49		1.62
0.03	0.97	0	0	1	0	0	0		1		1.553
0.07	0.93	0	0	1	0	0	0		1		1.54
0.17	0.83	0	0	1	0	0	0		1		1.56
0.05	0.95	0	0	1	0	0	0		1		1.53
0.07	0.93	0	0	1	0	0	0	0.15	2.85		1.62
0.15	0.85	0	0	1	0	0	0	0.15	2.85		1.62
0.3	0.7	0	0	1	0	0	0	0.15	2.85		1.62
0.45	0.55	0	0	1	0	0	0	0.15	2.85		1.62
0.15	0.85	0	0	1	0	0	0	0.3	2.7		1.55
0.2	0.8	0	0	1	0	0	0	1.05	1.95		1.76
0.25	0.75	0	0	1	0	0	0	0.9	2.1		1.73
0.2	0.8	0	0	1	0	0	0	0	1		1.59
0.085	0.915	0	0	1	0	0	0	0.45	2.55		1.625
0.13	0.87	0	0	1	0	0	0	0.39	2.61		1.56
0.1	0.9	0	0	1	0	0	0	0.9	2.1		1.7
0.1	0.9	0	0	1	0	0	0	0	1		1.53
0.15	0.85	0	0	1	0	0	0	0	1		1.54
0.25	0.75	0	0	1	0	0	0	0	1		1.54
0.3	0.7	0	0	1	0	0	0	0	1		1.49
0.1	0.9	0	0	1	0	0	0	0.09	2.91		1.54
0.1	0.9	0	0	1	0	0	0	0.6	2.4		1.61
1	0	0	0	1	0	0	0	0	1		1.6
0.5	0.5	0	0	1	0	0	0	0	1		1.63
0.75	0.25	0	0	1	0	0	0	0	1		1.66
0.2	0.8	0	0	1	0	0	0	1.5	1.5		1.82
0.17	0.83	0	0	1	0	0	0	0.81	2.19		1.7
0.17	0.83	0	0	1	0	0	0	0.75	2.25		1.75
0.15	0.85	0	0	1	0	0	0	0.9	2.1		1.72
1	1	0	0	1	0	0	0	0.2	2.8		1.65
1	1	0	0	1	0	0	0	0	1		1.51
1	1	0	0	1	0	0	0	1	1		1.69
1	1	0	0	1	0	0	0	1	1	1	1.8
0.15	0.85	0	0	1	0	0	0	0.87	2.13		1.72
0.05	0.95	0	0	1	0	0	0	0.15	2.85		1.55
0.07	0.93	0	0	1	0	0	0	0	1		1.53
0.4	0.6	0	0	1	0	0	0	0.095	0.905		1.61
0.3	0.7	0	0	1	0	0	0	0.095	0.905		1.63
0.2	0.8	0	0	1	0	0	0	0.095	0.905		1.65
0.1	0.9	0	0	1	0	0	0	0.095	0.905		1.56
0.4	0.6	0	0	1	0	0	0	0.256	0.744		1.65
0.3	0.7	0	0	1	0	0	0	0.256	0.744		1.72
0.2	0.8	0	0	1	0	0	0	0.256	0.744		1.71
0.1	0.9	0	0	1	0	0	0	0.256	0.744		1.72
0.17	0.83	0	0	1	0	0	0	0	1		1.51

0.17	0.83	0	0	0.999	0.001	0	0	0	1		1.51
0.17	0.83	0	0	0.995	0.005	0	0	0	1		1.52
0.17	0.83	0	0	0.99	0.01	0	0	0	1		1.52
0.17	0.83	0	0	0.98	0.02	0	0	0	1		1.51
0.17	0.83	0	0	0.95	0.05	0	0	0	1		1.52
0.17	0.83	0	0	0.9	0.1	0	0	0	1		1.47
0.17	0.83	0	0	0.8	0.2	0	0	0	1		1.36
0.17	0.83	0	0	0.7	0.3	0	0	0	1		1.32
0.17	0.83	0	0	0.6	0.4	0	0	0	1		1.28
0.17	0.83	0	0	0.5	0.5	0	0	0	1		1.26
0.17	0.83	0	0	0.4	0.6	0	0	0	1		1.25
0.17	0.83	0	0	0.3	0.7	0	0	0	1		1.25
0.15	0.85	0	0	1	0	0	0	0.45	2.55		1.59
0.88	0.12	0	0	1	0	0	0	0	1		1.51
0.17	0.83	0	0	1	0	0	0	1	2		1.67
1	1	0	0	1	0	0	0	0.3	2.7		1.56
1	1	0	0	1	0	0	0	1	0	1	2.32
0.1	0.9	0	0	1	0	0	0	0.1	2.9		1.55
0.08	0.92	0	0	1	0	0	0	0	1		1.51
0.88	0.12	0	0	1	0	0	0	0	1	0	1.5
0.05	0.788	0.162	0	1	0	0	0	0.51	2.49		1.59
0.05	0.81	0.14	0	1	0	0	0	0.45	2.55		1.56
0.05	0.78	0.13	0	1	0	0	0	0.45	2.55		1.6
0.05	0.75	0.2	0	1	0	0	0	0	1		1.52
0.025	0.475	0.5	0	0.5	0.5	0	0	0	1		1.27
0.05	0.45	0.5	0	0.5	0.5	0	0	0	1		1.26
0.1	0.76	0.14	0	1	0	0	0	0.51	2.49		1.61
0.05	0.81	0.14	0	0.95	0	0	0	0.43	2.42		1.64
0.05	0.79	0.16	0	1	0	0	0	0.5	2.5		1.57
0.05	0.79	0.16	0	0.75	0.25	0	0	0.5	2.5		1.34
0.05	0.79	0.16	0	0.5	0.5	0	0	0.5	2.5		1.31
0.05	0.79	0.16	0	0.25	0.75	0	0	0.5	2.5		1.3
0.1	0.75	0.15	0	1	0	0	0	0.5	2.5		1.59
0.1	0.75	0.15	0	0.75	0.25	0	0	0.5	2.5		1.36
0.1	0.75	0.15	0	0.5	0.5	0	0	0.5	2.5		1.31
0.1	0.75	0.15	0	0.25	0.75	0	0	0.5	2.5		1.29
0.2	0.66	0.14	0	1	0	0	0	0.5	2.5		1.6
0.2	0.66	0.14	0	0.75	0.25	0	0	0.5	2.5		1.38
0.2	0.66	0.14	0	0.5	0.5	0	0	0.5	2.5		1.31
0.2	0.66	0.14	0	0.25	0.75	0	0	0.5	2.5		1.28
0.05	0.8	0.15	0	1	0	0	0	0.15	2.85		1.617
0.05	0.79	0.16	0	1	0	0	0	0.51	2.49		1.58
0.05	0.83	0.12	0	1	0	0	0	0.39	2.61		1.6
0.05	0.8	0.15	0	1	0	0	0	0.45	2.55		1.63
0.1	0.81	0.09	0	1	0	0	0	0.1	2.9		1.55
0.07	0.785	0.115	0	1	0	0	0	0.45	2.55		1.57
0.05	0.76	0.19	0	1	0	0	0	0.6	2.4		1.59
0.2	0.2	0.6	0	1	0	0	0	0	1		1.64

0.1	0.2	0.7	0	1	0	0	0	0	1	1.62
0.3	0.2	0.5	0	1	0	0	0	0	1	1.64
0.4	0.2	0.4	0	1	0	0	0	0	1	1.64
0.08	0.76	0.15	0	1	0	0	0	0.51	2.49	1.62
0.12	0.83	0.05	0	1	0	0	0	1.2	1.8	1.74
0.05	0.5	0.45	0	0.5	0.5	0	0	0	1	1.22
0.1	0.75	0.15	0	1	0	0	0	1	2	1.73
0.05	0.79	0.16	0	1	0	0	0	1.2	2.6	1.71
0.05	0.38	0.57	0	1	0	0	0	0.2	2.8	1.57
0.1	0.36	0.54	0	1	0	0	0	0.2	2.8	1.59
0.2	0.32	0.48	0	1	0	0	0	0.2	2.8	1.607
0.05	0.79	0.16	0	1	0	0	0	0.45	2.55	1.63
0.05	0.79	0.16	0	1	0	0	0	0	1	1.55
0.04	0.82	0.14	0	1	0	0	0	0.42	2.58	1.58
0.05	0.76	0.19	0	1	0	0	0	0.57	2.32	1.69
0.03	0.945	0.025	0	1	0	0	0	0.075	2.925	1.56
0.08	0.55	0.37	0	1	0	0	0	0	1	1.53
0.05	0.79	0.16	0	1	0	0	0	0.48	2.52	1.61
0.1	0.75	0.24	0	1	0	0	0	0.51	2.49	1.61
0.05	0.79	0.16	0	1	0	0	0	1.5	1.5	1.85
0.1	0.75	0.15	0	1	0	0	0	0.51	2.49	1.63
0.13	0.72	0.16	0	1	0	0	0	0.51	2.49	1.63
0.5	0.75	0.1	0	1	0	0	0	0.51	2.49	1.63
0.2	0.75	0.05	0	1	0	0	0	0.51	2.49	1.63
0.05	0.83	0.12	0	1	0	0	0	0.51	2.49	1.63
0.1	0.83	0.07	0	1	0	0	0	0.51	2.49	1.63
0.14	0.83	0.03	0	1	0	0	0	0.51	2.49	1.63
0.05	0.68	0.26	0	0.75	0.25	0	0	0.4	2.6	1.36
0.01	0.39	0.6	0	1	0	0	0	0.025	2.975	1.6
0.02	0.38	0.6	0	1	0	0	0	0.025	2.975	1.6
0.03	0.37	0.6	0	1	0	0	0	0.025	2.975	1.6
0.5	0.4	0.1	0	1	0	0	0	0.51	2.49	1.7
0.07	0.77	0.16	0	1	0	0	0	0.5	2.5	1.639
0.04	0.8	0.16	0	1	0	0	0	0.5	2.5	1.632
0.03	0.81	0.16	0	1	0	0	0	0.5	2.5	1.63
0.05	0.7885	0.1615	0	1	0	0	0	0.3	2.7	1.55
0.2	0.664	0.336	0	1	0	0	0	1.05	1.95	1.77
0.1	0.1	0.8	0	1	0	0	0	0	1	1.5
0.05	0.85	0.1	0	1	0	0	0	0.45	2.45	1.59
0.05	0.7885	0.1615	0	1	0	0	0	0.51	2.49	1.57
0.05	0.57	0.38	0	1	0	0	0	0	1	1.54
0.1	0.54	0.36	0	1	0	0	0	0	1	1.51
0.025	0.825	0.15	0	1	0	0	0	0.45	2.55	1.651
0.05	0.79	0.16	0	1	0	0	0	2.4	2.49	1.63
0.07	0.73	0.2	0	1	0	0	0	0.47	2.53	1.6
0.1	0.74	0.153	0	1	0	0	0	0.51	2.49	1.61
0.16	0.8	0.04	0	1	0	0	0	1.5	1.5	1.82
0.16	0.8	0.04	0	1	0	0	0	1.71	1.29	1.89

0.16	0.8	0.04	0	1	0	0	0	1.83	1.17	1.91
0.15	0.65	0.2	0	1	0	0	0	0.6	2.4	1.68
0.05	0.7885	0.1615	0	1	0	0	0	0.4845	2.51555	1.6
0.07	0.79	0.14	0	1	0	0	0	0.45	2.55	1.6
0.05	0.8075	0.1425	0	1	0	0	0	0.45	2.55	1.64
0.05	0.49	0.16	0	1	0	0	0	0.51	2.49	1.73
0.1	0.747	0.153	0	1	0	0	0	0.51	2.49	1.73
0.05	0.7885	0.1441	0	1	0	0	0	0.3	2.7	1.55
0.05	0.7	0.25	0	1	0	0	0	0	1	1.51
0.05	0.79	0.16	0	1	0	0	0	0.31	2.7	1.59
0.05	0.81	0.14	0	1	0	0	0	0.4	2.6	1.61
0.08	0.78	0.14	0	1	0	0	0	0.42	2.58	1.61
0.05	0.76	0.16	0	1	0	0	0	0.49	2.51	1.6
0.79	0.16	0.1	0	1	0	0	0	1	2	1.73
0.15	0.71	0.14	0	1	0	0	0	0.9	2.1	1.7
0.05	0.85	0.1	0	1	0	0	0	0.03	2.97	1.598
0.1	0.81	0.09	0	1	0	0	0	0.03	2.97	1.6
0.15	0.76	0.09	0	1	0	0	0	0.03	2.97	1.599
0.2	0.72	0.08	0	1	0	0	0	0.03	2.97	1.62
0.12	0.5	0.38	0	1	0	0	0	0.96	2.04	1.72
0.08	0.78	0.14	0	1	0	0	0	0.42	2.38	1.61
0.1	0.765	0.135	0	1	0	0	0	0.45	2.55	1.611
0.07	0.78	0.15	0	1	0	0	0	0.45	2.55	1.61
0.05	0.65	0.3	0	1	0	0	0	0.15	2.85	1.55
0.06	0.79	0.15	0	1	0	0	0	0.45	2.55	1.64
0.04	0.8	0.16	0	1	0	0	0	0.49	2.51	1.61
0.1	0.75	0.15	0	1	0	0	0	0.45	2.55	1.63
0.3	0.6	0.1	0	1	0	0	0	0.095	0.905	1.65
0.2	0.6	0.2	0	1	0	0	0	0.095	0.905	1.64
0.1	0.6	0.3	0	1	0	0	0	0.095	0.905	1.64
0.2668	0.666	0.0664	0	1	0	0	0	0.095	0.905	1.65
0.0664	0.666	0.2668	0	1	0	0	0	0.095	0.905	1.63
0.133	0.733	0.133	0	1	0	0	0	0.095	0.905	1.65
0.0664	0.8668	0.0664	0	1	0	0	0	0.095	0.905	1.63
0.3	0.6	0.1	0	1	0	0	0	0.256	0.744	1.72
0.2	0.6	0.2	0	1	0	0	0	0.256	0.744	1.7
0.1	0.6	0.3	0	1	0	0	0	0.256	0.744	1.7
0.2668	0.666	0.0664	0	1	0	0	0	0.256	0.744	1.71
0.0664	0.666	0.2668	0	1	0	0	0	0.256	0.744	1.71
0.133	0.733	0.133	0	1	0	0	0	0.256	0.744	1.71
0.0664	0.8668	0.0664	0	1	0	0	0	0.256	0.744	1.7
0.05	0.83	0.17	0	1	0	0	0	0	1	1.56
0.05	0.07	0.25	0	1	0	0	0	0	1	1.51
0.05	0.81	0.14	0	1	0	0	0	2.4	0.45	1.59
0.03	0.77	0.2	0	1	0	0	0	0.46	2.54	1.5
0.05	0.85	0.15	0	1	0	0	0	0.45	2.55	1.612
0.1	0.85	0.15	0	1	0	0	0	0.45	2.55	1.611
0.09	0.58	0.33	0	1	0	0	0	0.65	2.35	1.59

0.05	0.79	0.16	0	1	0	0	0	0.3	2.7		1.57
0.05	0.83	0.12	0	1	0	0	0	0.36	2.64		1.61
0.05	0.79	0.16	0	1	0	0	0	1	2		1.61
0.05	0.81	0.14	0	1	0	0	0	0.42	2.58		1.61
0.1	0.76	0.14	0	1	0	0	0	0.55	2.55		1.61
0.05	0.79	0.16	0	1	0	0	0	0.39	2.61		1.59
0.05	0.8	0.15	0	1	0	0	0	0.55	2.55		1.61
0.1	0.75	0.15	0	1	0	0	0	0.55	2.55		1.63
0.2	0.65	0.15	0	1	0	0	0	0.55	2.55		1.85
0.05	0.79	0.16	0	1	0	0	0	0.47	2.53		1.59
0.17	0.75	0.08	0	1	0	0	0	0.39	2.61		1.61
0.05	0.81	0.14	0	1	0	0	0	0.39	2.61		1.59
0.04	0.8	0.16	0	1	0	0	0	0.51	2.49		1.61
0.05	0.83	0.12	0	1	0	0	0	0.49	2.51		1.6
0.05	0.79	0.16	0	1.1	0	0	0	0.51	2.49		1.59
0.05	0.83	0.17	0	1	0	0	0	0.51	2.49		1.6
0.05	0.85	0.1	0	1	0	0	0	1	0		2.28
0.06	0.79	0.15	0	1	0	0	0	1.8	1.2		1.83
0.05	0.79	0.16	0	1	0	0	0	0.52	2.48		1.38
0.05	0.79	0.16	0	0.916	0.084	0	0	0.52	2.48		1.38
0.05	0.79	0.16	0	0.832	0.168	0	0	0.52	2.48		1.38
0.05	0.79	0.16	0	0.748	0.252	0	0	0.52	2.48		1.38
0.05	0.79	0.16	0	0.664	0.336	0	0	0.52	2.48		1.38
0.05	0.79	0.16	0	0.58	0.42	0	0	0.52	2.48		1.38
0.05	0.79	0.16	0	0.84	0.84	0	0	0.52	2.48		1.38
0.06	0.78	0.16	0	1	0	0	0	0.51	2.49		1.63
0.15	0.75	0.1	0	1	0	0	0	0.1	2.9		1.57
0.15	0.75	0.1	0	1	0	0	0	0	1		1.554
0.05	0.94	0.01	0	1	0	0	0	0.03	2.97		1.55
0.05	0.55	0.4	0	1	0	0	0	2.4	0.12		1.56
0.17	0.83	0	0	1	0	0	0	2.4	0.51		1.64
0.05	0.81	0.14	0	1	0	0	0	0.45	2.55	0	1.58
1	0	0	0	1	0	0	0	1.85	1.15	0	2.08
0.01	0	0.99	0	1	0	0	0	0.03	2.97	0	1.6
0.02	0	0.98	0	1	0	0	0	0.06	2.94	0	1.6
0.03	0	0.97	0	1	0	0	0	0.09	2.91	0	1.6
0.05	0	0.95	0	1	0	0	0		1		1.51
0.1	0	0.9	0	1	0	0	0		1		1.54
0.2	0	0.8	0	1	0	0	0		1		1.57
0.3	0	0.7	0	1	0	0	0		1		1.58
0.4	0	0.6	0	1	0	0	0		1		1.62
0.6	0	0.4	0	1	0	0	0		1		1.68
0.8	0	0.2	0	1	0	0	0		1		1.72
0.15	0	0.85	0	1	0	0	0		1		1.59
0.15	0	0.85	0	1	0	0	0	0.45	2.55		1.78
0.2	0	0.8	0	0.5	0.5	0	0		1		1.3
0.3	0	0.7	0	0.5	0.5	0	0		1		1.28
0.1	0	0.9	0	0.5	0.5	0	0		1		1.6

0.1	0	0.9	0	0.7	0.255	0	0		1		1.28
0.1	0	0.9	0	0.25	0.75	0	0		1		1.2
0.05	0	0.95	0	0	1	0	0		1		1.61
0.09	0	0.91	0	1	0	0	0		1		1.59
0.1	0	0.9	0	0.75	0.25	0	0		1		1.28
0.1	0	0.9	0	0	1	0	0	1.2	1.8		1.82
0.15	0	0.85	0	1	0	0	0	1.2	1.8		1.82
0.05	0	0.95	0	1	0	0	0	1.2	1.8		1.82
0.11	0	0.89	0	1	0	0	0		1		1.62
0.17	0	0.83	0	1	0	0	0		1		1.633
0.23	0	0.77	0	1	0	0	0		1		1.644
0.34	0	0.66	0	1	0	0	0		1		1.652
0.1	0	0.9	0	1	0	0	0	1	2		1.77
0.05	0.17	0.76	0		1	0	0	0	1	2	0	1.72
0	0	0	1	1	0	0	0	1	0.8	0	1.72
0	1	0	0	1	0	0	0		1		1.48
0	1	0	0	1	0	0	0	1			2.26
0	1	0	0	0.75	0.25	0	0		1		1.47
0	1	0	0	0.5	0.5	0	0		1		1.57
0	1	0	0	0.25	0.75	0	0	1	0	0	2.273
0	1	0	0	1	0	0	0	0	1		1.45
0	1	0	0	0	1	0	0		1		1.36
0	1	0	0	0	1	0	0	0.24	2.76		1.47
0	1	0	0	0	1	0	0	0.5	2.5		1.53
0	1	0	0	0	1	0	0	0.75	2.25		1.63
0	1	0	0	0	1	0	0	1	2		1.7
0	1	0	0	1	0	0	0	0.6	2.4		1.57
0	1	0	0	0.875	0.125	0	0		1		1.33
0	1	0	0	0.625	0.375	0	0		1		1.24
0	1	0	0	0.375	0.625	0	0		1		1.21
0	1	0	0	0.25	0.75	0	0		1		1.21
0	1	0	0	0.125	0.875	0	0		1		1.25
0	1	0	0	0.9	0.1	0	0		1		1.43
0	1	0	0	0.7	0.3	0	0		1		1.33
0	1	0	0	0.4	0.6	0	0		1		1.29
0	1	0	0	0.2	0.8	0	0		1		1.29
0	1	0	0	0	1	0	0	1			1.6
0	1	0	0	1	0	0	0	0.15	2.85		1.62
0	1	0	0	1	0	0	0	0.5	2.5		1.965
0	1	0	0	1	0	0	0	1	2		2.268
0	1	0	0	1	0	0	0	1.5	1.5		1.91
0	1	0	0	1	0	0	0	2	1		1.969
0	1	0	0	1	0	0	0	2.4	0.5		2.139
0	1	0	0	1	0	0	0	1	1		1.47
0	1	0	0		0.25	0	0		1		1.4
0	1	0	0		1	0	0		1		1.43
0	1	0	0	1	0	0	0	2.4	0	0.33	2.3
0	1	0	0	1	0	0	0	2.4	0	0.57	2.36

0	1	0	0	1	0	0	0	2.25	0	0.75	2.39
0	1	0	0	1	0	0	0	2.1	0	0.9	2.44
0	1	0	0	1	0	0	0	2	0	1	2.48
0	1	0	0	1	0	0	0	0.095	0.905		1.59
0	1	0	0	1	0	0	0	0.256	0.744		1.7
0	0.1	0.9	0	1	0	0	0	0.3	2.9		1.592
0	0.2	0.8	0	1	0	0	0	0.3	2.9		1.59
0	0.3	0.7	0	1	0	0	0	0	1		1.62
0	0.83	0.17	0	1	0	0	0	0.5	2.5		1.61
0	0.83	0.17	0	1	0	0	0	0.5	2.51		1.6
0	0.5	0.5	0	1	0	0	0		1		1.25
0	0.6	0.4	0	1	0	0	0		1		1.25
0	0.15	0.85	0	0.5	0.5	0	0	2.4	0.45		1.53
0	0.85	0.15	0	0.4	0.6	0	0	0.45	2.55		1.6
0	0.83	0.17	0	1	0	0	0	0.51	2.49		1.6
0	0.9	0.1	0	1	0	0	0	0.1	2.9		1.52
0	0.85	0.15	0	1	0	0	0	0.45	2.55		1.5
0	0.75	0.25	0	1	0	0	0	0.75	2.25		1.61
0	0.4	0.6	0	1	0	0	0	0.2	2.8		1.5
0	0.1	0.9	0	1	0	0	0		1		1.59
0	0.2	0.8	0	1	0	0	0		1		1.57
0	0.3	0.7	0	1	0	0	0		1		1.55
0	0.4	0.6	0	1	0	0	0		1		1.54
0	0.7	0.3	0	1	0	0	0		1		1.503
0	0.6	0.4	0	1	0	0	0		0.4		1.25
0	0.83	0.17	0	1	0.6	0	0	0.51	2.49		1.61
0	0.95	0.05	0	1	0	0	0	0.15	2.85		1.55
0	0.75	0.25	0	0.5	0.5	0	0		1		1.36
0	0.1	0.9	0	0	1	0	0		1		1.55
0	0.8	0.2	0	1	0	0	0	0.6	2.4		1.59
0	0.1	0.9	0	1	0	0	0	2.4	0.3		2.14
0	0.3	0.7	0	0.7	0.3	0	0	2.1	0.9		2.03
0	0.5	0.5	0	1	0	0	0	1.5	1.5		1.82
0	0.7	0.3	0	1	0	0	0	0.9	2.1		1.71
0	0.9	0.1	0	1	0	0	0	0.3	2.7		1.8
0	0.85	0.15	0	1	0	0	0	0.21	2.79		1.56
0	1	1	0	1	0	0	0		1		1.5
0	0.96	0.04	0	1	0	0	0	0.12	2.88		1.48
0	0.975	0.025	0	1	0	0	0		1		1.48
0	0.25	0.75	0	0	1	0	0		1		1.33
0	0.5	0.5	0	0	1	0	0		1		1.6
0	0.83	0.17	0	0	1	0	0	0.39	2.61		1.6
0	0.02	0.98	0	1	0	0	0		1		1.51
0	0.05	0.95	0	1	0	0	0		1		1.52
0	0.15	0.85	0	1	0	0	0		1		1.35
0	0.93	0.03	0	1	0	0	0	0.09	2.91		1.55
0	0.84	0.16	0	1	0	0	0	0.5	1.5		1.83
0	0.83	0.17	0	1	0	0	0		1		1.54

0	0.9	0.1	0	1	0	0	0		1	1.53
0	0.125	0.875	0	1	0	0	0		1	1.55
0	0.125	0.875	0	1	0	0	0	1	2	1.71
0	0.92	0.08	0	1	0	0	0	0.24	2.76	1.56
0	0.85	0.15	0	1	0	0	0		1	1.53
0	0.75	0.25	0	0	1	0	0		1	1.4
0	0.75	0.25	0	0	0.95	0	0		1	1.4
0	0.75	0.25	0	0	0.91	0	0		1	1.42
0	0.75	0.25	0	0	0.8	0	0		1	1.53
0	0.3	0.7	0	1	0	0	0	0.3	2.7	1.59
0	0.83	0.17	0	1	0	0	0	1.2	1.8	1.72
0	0.83	0.17	0	1	0	0	0	0.46	2.54	1.57
0	0.67	0.33	0	1	0	0	0	0.5	2.5	1.638
0	0.83	0.17	0	1	0	0	0	1.5	1.5	1.84
0	0.75	0.25	0	1	0	0	0	0.51	2.49	1.65
0	0.25	0.75	0	0.75	0.25	0	0	0	1	1.34
0	0.5	0.5	0	0.75	0.25	0	0	0	1	1.33
0	0.75	0.25	0	0.75	0.25	0	0	0	1	1.33
0	0.6	0.4	0	0.6	0.6	0	0	0	1	1.25
0	0.5	0.5	0	0.5	0.5	0	0	0	1	1.2
0	0.6	0.4	0	0.4	0.6	0	0	0	1	1.27
0	0.6	0.4	0	0.6		0	0	0	1	1.25
0	0.6	0.4	0	0.4	0.6	0	0	0.12	2.88	1.26
0	0.6	0.4	0	0.4	0.6	0	0	0.18	2.82	1.272
0	0.6	0.4	0	0.4	0.6	0	0	0.24	2.76	1.28
0	0.6	0.4	0	0.4	0.6	0	0	0.48	2.52	1.32
0	0.6	0.4	0	0.4	0.6	0	0	0.3	2.7	1.36
0	0.4	0.6	0	1	0	0	0	0	1	1.6
0	0.4	0.6	0	1	0	0	0	0.025	2.975	1.6
0	0.83	0.17	0	1	0	0	0	1	2	1.684
0	0.83	0.17	0	1	0	0	0	2	1	1.976
0	0.83	0.17	0	1	0	0	0	2.4	0.5	2.113
0	0.83	0.17	0	1	0	0	0	1	0	2.278
0	0.67	0.33	0	1	0	0	0	0	1	1.528
0	0.67	0.33	0	1	0	0	0	1	2	1.682
0	0.67	0.33	0	1	0	0	0	1.5	1.5	1.848
0	0.67	0.33	0	1	0	0	0	2	1	1.969
0	0.67	0.33	0	1	0	0	0	2.4	0.5	2.115
0	0.67	0.33	0	1	0	0	0	1	0	2.28
0	0.5	0.5	0	1	0	0	0	0	1	1.546
0	0.5	0.5	0	1	0	0	0	0.5	2.5	1.612
0	0.5	0.5	0	1	0	0	0	1	2	1.751
0	0.5	0.5	0	1	0	0	0	2	1	1.957
0	0.5	0.5	0	1	0	0	0	2.4	0.5	2.141
0	0.5	0.5	0	1	0	0	0	1	0	2.28
0	0.17	0.83	0	1	0	0	0	0	1	1.588
0	0.17	0.83	0	1	0	0	0	0.5	2.5	1.625
0	0.17	0.83	0	1	0	0	0	1	2	1.764

0	0.17	0.83	0	1	0	0	0	1.5	1.5	1.858
0	0.17	0.83	0	1	0	0	0	2	1	1.989
0	0.17	0.83	0	1	0	0	0	2.4	0.5	2.153
0	0.17	0.83	0	1	0	0	0	1	0	2.296
0	0.81	0.19	0	1	0	0	0	0.54	2.46	1.62
0	0.82	0.18	0	1	0	0	0	0.53	2.47	1.64
0	0.83	0.17	0	1	0	0	0	0.52	2.48	1.64
0	0.84	0.16	0	1	0	0	0	0.51	2.49	1.64
0	0.84	0.16	0	1	0	0	0	0.5	2.5	1.65
0	0.85	0.15	0	1	0	0	0	0.5	2.5	1.65
0	1	1	0	1	0	0	0	0	1	1.546
0	0.75	0.25	0	0	1	0	0	0	1	1.33
0	0.4	0.6	0	1	0	0	0	0.1	2.9	1.51
0	0.75	0.25	0	1	0	0	0	0.45	2.55	1.62
0	0.71	0.29	0	1	0	0	0	0.42	2.58	1.62
0	0.66	0.34	0	1	0	0	0	0.42	2.58	1.62
0	0.85	0.15	0	1	0	0	0	0	1	1.56
0	0.6	0.4	0	1	0	0	0	0	1	1.54
0	0.25	0.75	0	1	0	0	0	0	1	1.54
0	0.75	0.25	0	1	0	0	0	0	1	1.53
0	1	1	0	1	0	0	0	1	0	1.6
0	0.85	0.15	0	1	0	0	0	0.15	0.85	1.6
0	0.83	0.17	0	1	0	0	0	0.51	2.59	1.6
0	0.83	0.17	0	1	0	0	0	0.49	2.51	1.6
0	0.9	0.1	0	1	0	0	0	0	1	1.54
0	0.44	0.56	0	1	0	0	0	0.33	2.67	1.62
0	0.66	0.34	0	1	0	0	0	0	1	1.533
0	0.25	0.75	0	0	1	0	0	0	1	1.36
0	0.5	0.5	0	0	1	0	0	0	1	1.38
0	0.85	0.15	0	1	0	0	0	0.6	2.4	1.616
0	0.1	0.9	0	1	0	0	0	0	1	1.58
0	0.15	0.85	0	1	0	0	0	0	1	1.63
0	0.976	0.024	0	1	0	0	0	0.075	2.925	1.54
0	0.84	0.16	0	1	0	0	0	0.4	2.6	1.53
0	0.83	0.17	0	1	0	0	0	0.47	2.53	1.61
0	0.87	0.13	0	1	0	0	0	0	1	1.5
0	0.92	0.08	0	1	0	0	0	0	1	1.55
0	0.8	0.2	0	1	0	0	0	0	1	1.35
0	0.83	0.17	0	1	0	0	0	0.03	2.97	1.6
0	0.9	0.1	0	1	0	0	0	0.03	2.97	1.581
0	0.83	0.17	0	1	0	0	0	0.33	2.67	1.59
0	0.1	0.9	0	0.9	0.1	0	0	0	1	1.44
0	0.33	0.66	0	0.66	0.33	0	0	0	1	1.3
0	1	1	0	1	0	0	0	1	1	1.96
0	0.88	0.12	0	1	0	0	0	0	1	1.53
0	0.6	0.4	0	1	0	0	0	0.095	0.905	1.64
0	0.7	0.3	0	1	0	0	0	0.095	0.905	1.63
0	0.8	0.2	0	1	0	0	0	0.095	0.905	1.61

0	0.9	0.1	0	1	0	0	0	0.095	0.905		1.61
0	0.6	0.4	0	1	0	0	0	0.256	0.744		1.72
0	0.7	0.3	0	1	0	0	0	0.256	0.744		1.71
0	0.8	0.2	0	1	0	0	0	0.256	0.744		1.72
0	0.9	0.1	0	1	0	0	0	0.256	0.744		1.7
0	0.05	0.95	0	1	0	0	0	0	1		1.5
0	0.85	0.15	0	1	0	0	0	0.55	2.55		1.58
0	0.026	0.974	0	1	0	0	0	0	1		1.516
0	1		0	1	0	0	0	0.45	2.55		2.23
0	0.48	0.52	0	1	0	0	0	0	1		1.62
0	0.8	0.2	0	1	0	0	0	0.22	1		1.53
0	0.4	0.6	0	1	0	0	0	0.6	2.4		1.68
0	0.6	0.4	0	1	0	0	0	0.4	2.6		1.58
0	0.8	0.2	0	1	0	0	0	0.2	2.8		1.53
0	0.7	0.3	0	1	0	0	0	0.1	2.9		1.58
0	0.65	0.35	0	1	0	0	0	0	1		1.52
0	0.3	0.7	0	1	0	0	0	0.15	2.85		1.6
0	0.3	0.7	0	1	0	0	0	0.45	2.55		1.66
0	0.3	0.7	0	1	0	0	0	0.6	2.4		1.69
0	1.5	0	0.5	1	0	0	0	0	1		1.57
0	0.5	0	1.5	1	0	0	0	0	1		1.57
0	0	1	0	1	0	0	0	0	1		1.6
0	0	1	0	1	0	0	0	1	0		2.2
0	0	1	0	1	0	0	0	0.09	2.91		1.61
0	0	1	0	1	0	0	0	0.3	2.7		1.64
0	0	1	0	1	0	0	0	0.6	2.4		1.7
0	0	1	0	1	0	0	0	1.8	1.2		1.92
0	0	1	0	1	0	0	0	0.5	2.5		1.7
0	0	1	0	1	0	0	0	0.8	2.2		1.75
0	0	1	0	1	0	0	0	1.2	1.8		1.83
0	0	1	0	1	0	0	0	0.3	2.9		1.589
0	0	1	0	0	1	0	0	0	1		1.23
0	0	1	0	1	0	2	0	1	2		1.75
0	0	1	0	1	0	2	0	2	1		2.02
0	0	1	0	0.75	0.25	2	0	0	1		1.35
0	0	1	0	1	0	0	0	0.32	2.68		1.64
0	0	1	0	1	0	0	0	0.56	2.44		1.68
0	0	1	0	1	0	0	0	1.5	1.5		1.83
0	0	1	0	1	0	0	0	0.1	2.9		1.6
0	0	1	0	1	0	0	0	0.2	2.8		1.64
0	0	1	0	1	0	0	0	0.25	2.75		1.65
0	0	1	0	1	0	0	0	0.7	2.3		1.68
0	0	1	0	1	0	0	0	0.03	2.97		1.6
0	0	1	0	1	0	0	0	0.075	2.95		1.6
0	0	1	0	1	0	0	0	0.15	2.85		1.6
0	0	1	0	1	0	0	0	0.225	2.775		1.6
0	0	1	0	1	0	0	0	0.75	2.25		1.7
0	0	1	0	1	0	0	0	0.9	2.1		1.75

0	0	1	0	0.992	0	0	0	0	1		1.56
0	0	1	0	0.984	0	0	0	0	1		1.56
0	0	1	0	0.969	0	0	0	0	1		1.56
0	0	1	0	0.937	0	0	0	0	1		1.56
0	0	1	0	0	0	0	0	0	1	2	1.41
0	0	1	0	0.75	0.25	0	0	0	1	0	1.24
0	0	1	0	0	1	0	0	0	1	0	1.27
0	0	1	0	1	0	0	0	0	1	0	1.4
0	0	1	0	0	1	0	0	2	1	0	1.81
0	0	1	0	0	1	0	0	1.8	1	0.2	1.87
0	0	1	0	0	1	0	0	1.5	1	0.5	1.97
0	0	1	0	0	1	0	0	1	2	0	1.49
0	0	1	0	0	1	0	0	0.6	2.4	0	1.36
0	0	1	0	1	0	0	0	0.12	2.88	0	1.6
0	0	1	0	1	0	0	0	0.33	2.67	0	1.62
0	0	1	0	0	1	0	0	0	0	1	2.2
0	0	1	0	0	1	0	0	0.6	0	2.4	2.83
0	0	1	0	1	0	0	0	2	1	0	1.85
0	0	1	0	1	0	0	0	1	0	0	2
0	0	1	0	1	0	0	0	1.16	1.74	0	1.9
0	0	1	0	1	0	0	0	1	2	0	1.6
0	0	1	0	0	0	0	1	0	2		1.52
0	0	1	0	0.5	0.5	0	0	0.6	2.4		1.35
0	0	1	0	1	0	0	0	0.45	2.55		1.65
0	0	1	0	0	1	0	0	0.42	2.58		1.4
0	0	1	0	0	1	0	0	1	2		1.46
0	0	1	0	0	1	0	0	1.5	1.5		1.57
0	0	1	0	0	1	0	0	2	1		1.75
0	0	1	0	0	1	0	0	2.4	0.36		1.83
0	0	1	0	1	0	0	0	0.05	2.95		1.57
0	0	1	0	1	0	0	0	0.08	2.92		1.6
0	0	1	0	1	0	0	0	0.18	2.82		1.604
0	0	1	0	1	0	0	0	0.39	2.61		1.641
0	0	1	0	1	0	0	0	0.87	2.13		1.72
0	0	1	0	1	0	0	0	1.14	1.86		1.777
0	0	1	0	1	0	0	0	1.41	1.59		1.791
0	0	1	0	1	0	0	0	1.74	1.26		1.924
0	0	1	0	1	0	0	0	2.13	0.87		2.033
0	0	1	0	1	0	0	0	2.4	0.48		2.154
0	0	1	0	0.8	0.2	0	0	0	1		1.5
0	0	1	0	0.6	0.4	0	0	0	1		1.39
0	0	1	0	0.4	0.6	0	0	0	1		1.27
0	0	1	0	0.2	0.8	0	0	0	1		1.189
0	0	1	0	0.75	0.25	0	0	1.2	1.8		1.59
0	0	1	0	0.5	0.5	0	0	1.2	1.8		1.48
0	0	1	0	0.25	0.75	0	0	1.2	1.8		1.34
0	0	1	0	0	1	0	0	1.2	1.8		1.6
0	0	1	0	0.75	0.25	0	0	0.3	2.7		1.43

0	0	1	0	0.75	0.25	0	0	0.6	2.4	1.48
0	0	1	0	0.75	0.25	0	0	0.9	2.1	1.53
0	0	1	0	0.75	0.25	0	0	1.5	1.5	1.65
0	0	1	0	0.75	0.25	0	0	1.8	1.2	1.73
0	0	1	0	0.75	0.25	0	0	2.1	0.9	1.8
0	0	1	0	0.75	0.25	0	0	2.4	0.6	1.9
0	0	1	0	0.75	0.25	0	0	2.4	0.3	1.99
0	0	1	0	0.75	0.25	0	0	1	0	2.03
0	0	1	0	0	1	0	0	1	0	2.15
0	0	1	0	0.7	0.3	0	0	0	1	1.2
0	0	1	0	0.5	0.5	0	0	0	1	1.18
0	0	1	0	0.3	0.7	0	0	0	1	1.17
0	0	1	0	0.25	0.75	0	0	0	1	1.17
0	0	1	0	0.5	0.5	0	0		1	1.3
0	0	1	0	0.25	0.75	0	0		1	1.25
0	0	1	0	0.7	0.3	0	0		1	1.37
0	0	1	0	0.38	0.62	0	0		1	1.22
0	0	1	0	0.2	0.8	0	0		1	1.22
0	0	1	0	0	1	0	0		1	1.28
0	0	1	0	0	0.19	0	0		1	1.31
0	0	1	0	0.9	0.1	0	0		1	1.31
0	0	1	0	0.7	0.255	0	0		1	1.24
0	0	0.75	0	0.25	0	0	0		1	1.38
0	0	1	0	0.875	0.125	0	0		1	1.42
0	0	1	0	0.75	0.25	0	0		1	1.31
0	0	1	0	0.625	0.375	0	0		1	1.25
0	0	1	0	0.375	0.625	0	0		1	1.2
0	0	1	0	0.125	0.875	0	0		1	1.18
0	0	1	0	0.85	0.15	0	0		1	1.37
0	0	1	0	0.65	0.35	0	0		1	1.25
0	0	1	0	0.4	0.6	0	0	1	1	1.16
0	0	1	0	0.4	0.6	0	0	0.6	2.4	1.24
0	0	1	0	0.4	0.6	0	0	0.9	2.1	1.25
0	0	1	0	0.4	0.6	0	0	1.2	1.8	1.26
0	0	1	0	0.4	0.6	0	0	1.5	1.5	1.27
0	0	1	0	0.4	0.6	0	0	1.8	1.2	1.29
0	0	1	0	0.4	0.6	0	0	2.1	0.9	1.3
0	0	1	0	0.4	0.6	0	0	2.4	0.6	1.32
0	0	1	0	0.85	0.15	0	0	0	1	1.32
0	0	1	0	0.7	0.255	0	0	0	1	1.3
0	0	1	0	0.4	0.6	0	0	0.4	2.6	1.26
0	0	1	0	0.2	0.8	0	0	0.4	2.6	1.26
0	0	1	0	0.6	0.4	0	0	0.4	2.6	1.26
0	0	1	0	0.8	0.2	0	0	0.4	2.6	1.26
0	0	1	0	0.95	0.05	0	0	0	1	1.5
0	0	1	0	0.9	0.1	0	0	0	1	1.5
0	0	1	0	1	0	0	0	0.033	2.97	1.6
0	0	1	0	1	0	0	0	0.066	2.93	1.61

0	0	1	0	1	0	0	0	0.083	2.92		1.61
0	0	1	0	1	0	0	0	2.4	0.5		2.161
0	0	1	0	1	0	0	0	1	1		1.594
0	0	1	0	1	0	0	0	0.4	2.6		1.746
0	0	1	0	1	0	0	0	2.25	0.75		1.67
0	0	1	0	0.97	0	0.03	0	0	1		1.58
0	0	1	0	0.95	0	0.05	0	0	1		1.63
0	0	1	0	0.92	0	0.08	0	0	1		1.65
0	0	1	0	0.9	0	0.1	0	0	1		1.67
0	0	1	0	1	0	0	0	0.075	2.925		1.61
0	0	1	0	1	0	0	0	0.36	2.64		1.62
0	0	1	0	1	0	0	0	0.78	2.22		1.69
0	0	1	0	1	0	0	0	1.77	1.23		1.96
0	0	1	0	1	0	0	0	2.4	0.15		2.23
0	0	1	0	1	0	0	0	1			2.27
0	0	1	0	1	0	0	0		1		1.67
0	0	1	0	0.95	0	0	0	0.1	2.9		1.55
0	0	1	0	0.9	0.05	0	0	0.1	2.9		1.55
0	0	1	0	0.85	0.1	0	0	0.1	2.9		1.44
0	0	1	0	0.8	0.15	0	0	0.1	2.9		1.39
0	0	1	0	1	0	0	0	0.9	1		1.72
0	0	1	0	1	0	0	0	1.25	0.75		1.76
0	0	1	0	1	0	0	0	0.11	2.89		1.585
0	0	1	0	1	0	0	0	0.14	2.86		1.591
0	0	1	0	1	0	0	0	0.33	2.67		1.65
0	0	0.98	0	1	0	0	0	0	1		1.59
0	0	1	0	1	0	0	0	0	2		2.1
0	0	1	0	1	0	0	0	1.7	1.3		1.94
0	0	1	0	1	0	0	0	0.81	2.19		1.71
0	0	1	0	0.98	0	0.02	0		1		1.5
0	0	1	0	0	0	0	0	2	0	1	2.12
0	0	1	0	0	0	0	0	1	0	0.5	1.8
0	0	1	0	0.95	0	0	0		1		1.632
0	0	1	0	0.9	0	0	0		1		1.63
0	0	1	0	0.8	0	0	0		1		1.628
0	0	1	0	1	0	0	0	0.02	2.98		1.6
0	0	1	0	0	1	0	0			1	2.5
0	0	1	0	0	1	0	0	0	2.6		1.85
0	0	1	0	0.97	0	0.03	0	2.4	0.09		1.59
0	0	1	0	0	0	0	0	1	0		1.38
0	0	1	0	0	0	0	0		1		1.65
0	0	1	0	0.9	0	0	0		1	0	1.61
0	0	1	0	0.5	0.5	0	0		1	0	1.21
0	0	1	0	1	0	0	0		1	0	1.605
0	0	1	0	1	0	0	0	0.4	2.6	0	1.67
0	0	1	0	1	0	0	0	0.2	2.8	0	1.63
0	0	1	0	1	0	0	0	0.66	2.33	0	1.58
0	0	1	0	1	0	0	0	0.33	2.66	0	1.65

	0		0	1			0		1	0	0	0	1.5	1.5	0	1.72
	0		0	1			0		1	0	0	0	0.15	2.85	0	1.57
	0		0	1			0		1	0	0	0	0.3	2.7	0	1.58
	0		0	1			0		1	0	0	0	0.45	2.55	0	1.6
	0		0	1			0		1	0	0	0	0.6	2.4	0	1.62
	0		0	1			0		1	0	0	0	0	0	1	2.8
	0		0	1			0		1	0	0	0	0	2	1	2.7
	0		0	1			0		0.93	0	0	0	0	1	0	1.44
	0		0	1			0		0	0	0	1	0	2	0	2.2
	0		0	1			0		1	0	0	0	2.1	0.9	0	1.97
	0		0	1			0		1	0	0	0	1.26	1.74	0	1.79
	0		0	1			0		1	0	0	0	2.16	0.84	0	2.01
	0		0	1			0		1	0	0	0	1.2	1.8	0	1.6
	0		0	1			0		1	0	0	0	0.9	2.1	0	1.6
1			0		0		0		0	0	0	0	0	1	0	1.81
	0	1			0		0		1	0	0	1		1		1.3
	0		1		0	1			1	0	0	0	0	1		1.72
	0		1		0	2			1	0	0	0	0	1		1.76
	0		0	1			0		1	0	2	0	1	0		2.24
	0		0	1			0		1	0	0	0	1	2		1.91
	0		0	1			0		1	0	0	0	2	1		2.35
	0		0	1			0		0.75	0.25	0	0	0	1		2.05
	0		0	1		1		1		0	0	0	0	1	0	1.68
	0		0	0.75		0.5			1	0	0	0	0	1	0	1.74
1			0		0		0		0	1	0	0	0	1	0	1.8
1			0		0		0		1	0	0	0	0	1	0	1.3
1			0		0		0		1	0	0	0	0	1	0	2.05
1			0		0		0		1	0	0	0	1	0	0	2.04
1			0		0		0		1	0	0	0	1	2	0	1.9
1			0		0		0		1	0	0	0	1	0	0	1.89
1			0		0		0		1	0	0	0	2	1	0	1.88
1			0		0		0		1	0	0	0	0	1	0	1.85
1			0		0		0		1	0	0	0	0	1	0	1.93
1			0		0		0		0	1	0	0	1	0	0	1.73
1			0		0		0		0	1	0	0	0	1	0	1.71
1			0		0		0		1	0	0	0	0	1	0	2.01
1			0		0		0		1	0	0	0	2	1	0	2.31
1			0		0		0		1	0	0	0	1	2	0	2.28
1			0		0		0		1	0	0	0	1	2	0	1.89
1			0		0		0		1	0	0	0	1	2	0	1.94
1			0		0		0		1	0	0	0	1	2	0	2.36
1			0		0		0		1	0	0	0	1	2	0	1.69
1			0		0		0		1	0	0	0	1	0	0	1.839
1			0		0		0		1	0	0	0	0	1	0	2.33
1			0		0		0		1	0	0	0	2	1	0	1.79
1			0		0		0		1	0	0	0	1	0	0	1.77
1			0		0		0		1	0	0	0	1	0	0	1.72
1			0		0		0		1	0	0	0	2	1	0	2.34

1		0	0	0	1	0	0	0	1	2	0	1.9
1		0	0	0	1	0	0	0	1	0	0	1.82
1		0	0	0	1	0	0	0	0	1	0	1.56
1		0	0	0	1	0	0	0	1	2	0	2.39
1		0	0	0	1	0	0	0	1	0	0	1.75
1		0	0	0	1	0	0	0	0.15	2.85	0	2.28
1		0	0	0	1	0	0	0	0	1	0	1.6
1		0	0	0	1	0	0	0	0	1	0	2.4
1		0	0	0	1	0	0	0	1	0	0	1.5
1		0	0	0	1	0	0	0	2	1	0	1.88
1		0	0	0	1	0	0	0	1	2	0	1.25
1		0	0	0	0.96	0	0	0.04	0	1	0	1.79
1		0	0	0	1	0	0	0	0	1	0	1.901
1		0	0	0	1	0	0	0	0	1	0	1.67
1		0	0	0	1	0	0	0	1	0	0	1.95
1		0	0	0	1	0	0	0	0	1	0	
1		0	0	0	1	0	0	0	1	0	0	1.97
1		0	0	0	1	0	0	0	0	1	0	1.68
1		0	0	0	1	0	0	0	2	1	0	2.24
1		0	0	0	0	1	0	0	0	1	0	2.58
1		0	0	0	0	1	0	0	1	0	0	2.1
1		0	0	0	1	0	0	0	1	2	0	1.906
1		0	0	0	1	0	0	0	0	1	0	1.8
1		0	0	0	1	0	0	0	1	2	0	1.87
1		0	0	0	1	0	0	0	1	2	0	2.26
	1	0	0	0	1	0	0	0	0	1	0	1.84
1		0	0	0	1	0	0	0	0	1	0	2.39
1		0	0	0	1	0	0	0	1	0	0	1.63
1		0	0	0	1	0	0	0	2	1	0	2.27
1		0	0	0	1	0	0	0	1	2	0	2.29
1		0	0	0	1	0	0	0	0	1	0	1.92
1		0	0	0	1	0	0	0	1	2	0	1.91
1		0	0	0	1	0	0	0	1	0	0	1.91
1		0	0	0	1	0	0	0	1	2	0	2.3
1		0	0	0	0	0	0	1	1	0	0	2.3
1		0	0	0	1	0	0	0	0	1	0	2.3
1		0	0	0	0.97	0	0	0	1	0	0	2.3
1		0	0	0	0.97	0	0	0	1	0	0	1.91
1		0	0	0	1	0	0	0	2	1	0	1.8
1		0	0	0	1	0	0	0	1	2	0	1.74
1		0	0	0	1	0	0	0	1	1	0	1.72
	1	0	0	0	1	0	0	0	1	0	0	1.74
1		0	0	0	1	0	0	0	1	1	0	1.63
1		0	0	0	1	0	0	0	1	2	0	1.8
	1	0	0	0	1	0	0	0	1	1	0	1.6
	1	0	0	0	1	0	0	0	2	1	0	1.32
	1	0	0	0	1	0	0	0	0	1	0	1.83
0.17	0.83		0	0	1	0	0	0	1.5	1.5		1.55

0.17	0.83	0	0	1	0	0	0	1.2	1.8	1.61
0.17	0.83	0	0	1	0	0	0	1.2	1.8	1.59
0.17	0.83	0	0	1	0	0	0	0.51	2.49	1.57
0.17	0.83	0	0	1	0	0	0	1.5	1.5	1.55
0.17	0.83	0	0	1	0	0	0	0.51	2.49	1.752
0.2	0.8	0	0	0.5	0.5	0	0		1	1.49
0.17	0.83	0	0	1	0	0	0	1.5	1.5	1.51
0.1	0.9	0	0	1	0	0	0		1	1.55
0.05	0.95	0	0	1	0	0	0	0	1	1.57
0.15	0.85	0	0	1	0	0	0	0.15	2.85	1.52
0.125	0.875	0	0	1	0	0	0	0.375	2.625	1.58
0.2	0.8	0	0	1	0	0	0	0	1	1.7
0.17	0.83	0	0	1	0	0	0	1.2	1.8	1.82
0.1	0.9	0	0	1	0	0	0	0	1	1.52
0.2	0.8	0	0	1	0	0	0	0	1	1.64
0.1	0.9	0	0	1	0	0	0	0.3	2.7	1.71
0.25	0.75	0	0	1	0	0	0	0	1	1.48
0.05	0.95	0	0	1	0	0	0	0	1	1.55
0.3	0.7	0	0	1	0	0	0	0	1	1.56
0.25	0.75	0	0	1	0	0	0	0.6	2.4	1.54
0.2	0.8	0	0	1	0	0	0	1.2	1.8	1.554
0.15	0.85	0	0	1	0	0	0	0	1	1.55
0.5	0.5	0	0	1	0	0	0	0	1	1.62
0.75	0.25	0	0	1	0	0	0	0	1	1.555
0.1	0.9	0	0	1	0	0	0	0	1	1.6
0.17	0.83	0	0	1	0	0	0	0	1	1.59
1	1	0	0	1	0	0	0	0	1	1.62
0.05	0.95	0	0	1	0	0	0	0	1	1.65
0.15	0.85	0	0	1	0	0	0	0	1	1.61
0.05	0.95	0	0	1	0	0	0	0	1	1.62
0.05	0.81	0.14	0	1	0	0	0	0.45	2.55	1.63
0.05	0.79	0.16	0	1	0	0	0	0.5	2.5	1.599
0.05	0.79	0.16	0	1	0	0	0	0.51	2.49	1.59
0.05	0.79	0.16	0	1	0	0	0	0.51	2.49	1.6
0.2	0.2	0.6	0	1	0	0	0	0	1	1.59
0.05	0.81	0.14	0	1	0	0	0	0.45	2.55	1.58
0.05	0.79	0.16	0	1	0	0	0	0.51	2.49	1.6
0.05	0.79	0.16	0	1	0	0	0	0.51	2.49	1.84
0.05	0.79	0.16	0	1	0	0	0	0.5	2.5	1.55
0.05	0.79	0.16	0	1	0	0	0	0.51	2.49	1.6
0.05	0.8	0.15	0	1	0	0	0	0.45	2.55	1.5
0.05	0.81	0.14	0	1	0	0	0	0.45	2.55	1.61
0.05	0.7885	0.1615	0	1	0	0	0	0.51	2.49	1.6
0.05	0.79	0.16	0	1	0	0	0	0.48	2.52	1.58
0.05	0.8	0.15	0	1	0	0	0	0.45	2.55	1.5
0.05	0.79	0.16	0	1	0	0	0	0.51	2.49	1.73
0.05	0.7885	0.1615	0	1	0	0	0	0.51	2.49	1.612
0.05	0.7885	0.1615	0	1	0	0	0	0.51	2.49	1.585

0.07	0.79	0.14	0	1	0	0	0	0.45	2.55	1.55
0.05	0.7885	0.1615	0	1	0	0	0	0.51	2.49	1.63
0.05	0.8075	0.1425	0	1	0	0	0	0.45	2.55	1.54
0.05	0.79	0.16	0	1	0	0	0	0.45	2.55	1.61
0.05	0.79	0.16	0	1	0	0	0	0.51	2.49	1.61
0.05	0.81	0.14	0	1	0	0	0	0.45	2.55	1.61
0.05	0.81	0.14	0	1	0	0	0	0.45	2.55	1.6
0.05	0.81	0.14	0	1	0	0	0	0.45	2.55	1.55
0.05	0.79	0.16	0	1	0	0	0	0.48	2.52	1.5
0.05	0.79	0.16	0	1	0	0	0	0.48	2.52	1.7
0.05	0.79	0.16	0	1	0	0	0	0	1	1.58
0.06	0.79	0.15	0	1	0	0	0	0.45	2.55	1.59
0.05	0.79	0.16	0	1	0	0	0	0.45	2.55	1.63
0.05	0.79	0.16	0	1	0	0	0	0	1	1.64
0.05	0.8	0.15	0	1	0	0	0	0.45	2.55	1.63
0.05	0.79	0.16	0	1	0	0	0	0.3	2.7	1.3
0.05	0.81	0.14	0	1	0	0	0	0.45	2.55	1.39
0.05	0.79	0.16	0	1	0	0	0	0.45	2.55	1.62
0.05	0.85	0.15	0	1	0	0	0	0.45	2.55	1.39
0.06	0.79	0.15	0	1	0	0	0	0.45	2.55	1.26
0.05	0.79	0.16	0	1	0	0	0	0.5	2.5	1.41
0.05	0.79	0.16	0	1	0	0	0	0.51	2.49	1.47
0.1	0.75	0.15	0	1	0	0	0	0.5	2.5	1.4
0.1	0	0.9	0	1	0	0	0		1	1.59
0.1	0	0.9	0	1	0	0	0		1	1.4
0.2	0	0.8	0	1	0	0	0		1	2.23
0.1	0	0.9	0	1	0	0	0		1	1.498
0.1	0	0.9	0	0.5	0.5	0	0		1	1.37
0.1	0	0.9	0	0.25	0.75	0	0		1	1.22
0	1	0	0	1	0	0	0		1	1.3
0	1	0	0	1	0	0	0		1	1.22
0	1	0	0	1	0	0	0		1	1.2
0	1	0	0	0	1	0	0		1	1.3
0	1	0	0	1	0	0	0	1		1.39
0	1	0	0	1	0	0	0		1	1.51
0	1	0	0	0.75	0.25	0	0		1	1.517
0	1	0	0	0.5	0.5	0	0		1	1.38
0	1	0	0	0	1	0	0		1	1.6
0	1	0	0	0.5	0.5	0	0		1	1.6
0	1	0	0	0	1	0	0		1	1.55
0	1	0	0	0.5	0.5	0	0		1	1.32
0	1	0	0	0	1	0	0		1	1.54
0	1	0	0	1	0	0	0	0	1	1.531
0	1	0	0	1	0	0	0	1		1.512
0	1	0	0	0	1	0	0		1	1.55
0	1	0	0	1	0	0	0		1	1.62
0	1	0	0	0	1	0	0	1	2	1.56
0	1	0	0	0	1	0	0		1	1.53

0	1		0	0	1	0	0	0		1	1.54
0	0.5	0.5		0	1	0	0	0		1	1.73
0	0.6	0.4		0	1	0	0	0		1	1.6
0	0.9	0.1		0	1	0	0	0	0.1	2.9	1.528
0	0.83	0.17		0	1	0	0	0	0.51	2.49	1.55
0	0.9	0.1		0	1	0	0	0	0.1	2.9	1.55
0	0.7	0.3		0	1	0	0	0		1	1.56
0	0.1	0.9		0	1	0	0	0		1	1.58
0	0.85	0.15		0	1	0	0	0	0.45	2.55	1.53
0	0.2	0.8		0	1	0	0	0		1	1.59
0	0.1	0.9		0	1	0	0	0		1	1.55
0	0.5	0.5		0	1	0	0	0		1	1.5
0	1	1		0	1	0	0	0		1	1.55
0	0.4	0.6		0	1	0	0	0	0.2	2.8	1.57
0	0.3	0.7		0	1	0	0	0		1	1.57
0	0.4	0.6		0	1	0	0	0		1	1.6
0	0.85	0.15		0	1	0	0	0	0.45	2.55	1.56
0	0.6	0.4		0	1	0	0	0		1	1.65
0	0.2	0.8		0	1	0	0	0		1	1.63
0	0.85	0.15		0	1	0	0	0	0.45	2.55	1.24
0	0.83	0.17		0	1	0	0	0	0.51	2.49	1.57
0	0.9	0.1		0	1	0	0	0	0.1	2.9	1.57
0	0.7	0.3		0	1	0	0	0		1	1.56
0	0.85	0.15		0	1	0	0	0	0.45	2.55	1.53
0	0.83	0.17		0	1	0	0	0	0.51	2.49	1.52
0	0.83	0.17		0	1	0	0	0	0.51	2.49	1.53
0	0.5	0.5		0	0.5	0.5	0	0	0	1	1.629
0	0.85	0.15		0	1	0	0	0	0.45	2.55	1.866
0	0.1	0.9		0	1	0	0	0		1	1.845
0	0.3	0.7		0	1	0	0	0		1	1.638
0	0.7	0.3		0	1	0	0	0		1	1.627
0	0.9	0.1		0	1	0	0	0		1	1.613
0	0.83	0.17		0	1	0	0	0		1	1.58
0	0.83	0.17		0	1	0	0	0	0.5	2.5	1.56
0	0.83	0.17		0	1	0	0	0	1.5	1.5	1.6
0	0.5	0.5		0	1	0	0	0	1.5	1.5	1.51
0	0.83	0.17		0	1	0	0	0	0.51	2.49	1.53
0	0.83	0.17		0	1	0	0	0	0.5	2.5	1.51
0	0.83	0.17		0	1	0	0	0	0.5	2.5	1.645
0	0.85	0.15		0	1	0	0	0	0.45	2.55	1.627
0	0.4	0.6		0	1	0	0	0	0.1	2.9	1.5
0	0.3	0.7		0	1	0	0	0	0	1	1.47
0	0.85	0.15		0	1	0	0	0	0.45	2.55	1.63
0	0.95	0.05		0	1	0	0	0	0.15	2.85	1.64
0	0.95	0.05		0	1	0	0	0	0.15	2.85	1.33
0	0.83	0.17		0	1	0	0	0	0.51	2.49	1.55
0	0.85	0.15		0	1	0	0	0	0.5	2.5	1.51
0	0.9	0.1		0	1	0	0	0	0.3	2.7	1.59

0	0.85	0.15	0	1	0	0	0	0.45	2.55		1.63
0	0.85	0.15	0	1	0	0	0	0.45	2.55		1.549
0	0.83	0.17	0	1	0	0	0	0.51	2.49		1.58
0	0.25	0.75	0	0.75	0.25	0	0	0	1		1.55
0	0.85	0.15	0	1	0	0	0	0	1		1.61
0	0.1	0.9	0	1	0	0	0	0	1		1.36
0	0.9	0.1	0	1	0	0	0	0.1	2.9		2.25
0	0.83	0.17	0	1	0	0	0	0.49	2.51		1.61
0	0.1	0.9	0	1	0	0	0	0	1		1.59
0	0.4	0.6	0	1	0	0	0	0.2	2.8		1.55
0	0.9	0.1	0	1	0	0	0	0	1		1.55
0	0.8	0.2	0	1	0	0	0	0.6	2.4		1.63
0	0.5	0.5	0	0.5	0.5	0	0	0	1		1.63
0	0.83	0.17	0	1	0	0	0	0.51	2.49		1.59
0	0.85	0.15	0	1	0	0	0	0.45	2.55		1.57
0	0.85	0.15	0	1	0	0	0	0.45	2.55		1.57
0	0.85	0.15	0	1	0	0	0	0.45	2.55		1.55
0	0.3	0.7	0	1	0	0	0	0	1		1.65
0	0.3	0.7	0	1	0	0	0	0.3	2.7		2.3
0	0.85	0.15	0	1	0	0	0	0.15	0.85		1.72
0	0.85	0.15	0	1	0	0	0	0.45	2.55		1.6
0	0	1	0	1	0	0	0	0	1		2.29
0	0	1	0	1	0	0	0	0.6	2.4		1.81
0	0	1	0	1	0	0	0	0.6	2.4		2.09
0	0	1	0	1	0	0	0	0.3	2.7		2.13
0	0	1	0	1	0	0	0	1	0		1.7
0	0	1	0	1	0	0	0	1	0		2.8
0	0	1	0	1	0	0	0	0	1		1.94
0	0	1	0	0	1	0	0	0	0	1	1.31
0	0	1	0	1	0	0	0	0	1	0	1.53
0	0	1	0	1	0	0	0	0	1		1.48
0	0	1	0	1	0	0	0	0.15	2.85		1.65
0	0	1	0	1	0	0	0	0	1		1.65
0	0	1	0	1	0	0	0	0.15	2.85		1.65
0	0	1	0	1	0	0	0	0.3	2.7		1.576
0	0	1	0	1	0	0	0	0.9	2.1		1.79
0	0	1	0	1	0	0	0	0	1		1.62
0	0	1	0	1	0	0	0	1	2		1.34
0	0	1	0	1	0	0	0	0.3	2.7		2.08
0	0	1	0	0	1	0	0	0	1		1.674
0	0	1	0	0	1	0	0	0	1		1.612
0	0	1	0	1	0	0	0	0.6	2.4		1.3
0	0	1	0	1	0	0	0	0	1		1.56
0	0	1	0	0	1	0	0	0	1		1.17
0	0	1	0	0	1	0	0	1	2		1.24
0	0	1	0	0.5	0.5	0	0	0	1		1.3
0	0	1	0	0.75	0.25	0	0	0	1		1.22
0	0	1	0	0.5	0.5	0	0	0	1		1.38

0	0	1	0	0.75	0.25	0	0	0	1		1.28
0	0	1	0	0.5	0.5	0	0		1		1.27
0	0	1	0	0.25	0.75	0	0		1		1.37
0	0	1	0	0	1	0	0		1		1.23
0	0	1	0	0.5	0.5	0	0		1		1.18
0	0	1	0	0.25	0.75	0	0		1		1.22
0	0	1	0	0	1	0	0		1		1.26
0	0	1	0	0.75	0.25	0	0		1		1.26
0	0	1	0	0.7	0.255	0	0		1		1.51
0	0	1	0	0.85	0.15	0	0	0	1		1.6
0	0	1	0	1	0	0	0	0.05	2.95		1.609
0	0	1	0	1	0	0	0	0	1		1.87
0	0	1	0	1	0	0	0	1.5	1.5		1.656
0	0	1	0	1	0	0	0	0.5	2.5		1.7
0	0	1	0	1	0	0	0	1	2		2.027
0	0	1	0	1	0	0	0	2	1		2.308
0	0	1	0	1	0	0	0	1	0		1.78
0	0	1	0	1	0	0	0	2	1		1.608
0	0	1	0	1	0	0	0	0	1		2.18
0	0	1	0	0	1	0	0	1	0		2.13
0	0	1	0	0	1	0	0	2	1		1.67
0	0	1	0	0	1	0	0	1.5	1.5		1.614
0	0	1	0	1	0	0	0	0.2	2.8		1.638
0	0	1	0	1	0	0	0	0.1	2.9		1.684
0	0	1	0	1	0	0	0	0.3	2.7		1.614
0	0	1	0	1	0	0	0	0.3	2.7		1.65
0	0	1	0	1	0	0	0	0.4	2.6		1.683
0	0	1	0	1	0	0	0	0.5	2.5		1.597
0	0	1	0	1	0	0	0	0	1		1.67
0	0	1	0	1	0	0	0	0.75	2.25		1.67
0	0	1	0	1	0	0	0	1.5	1.5		2.1
0	0	1	0	1	0	0	0	0	1		1.9
0	0	1	0	1	0	0	0	1.5	1.5		1.77
0	0	1	0	1	0	0	0	1.2	1.8		1.77
0	0	1	0	1	0	0	0	1	2		1.71
0	0	1	0	1	0	0	0	0.87	2.13		1.66
0	0	1	0	1	0	0	0	0.6	2.4		1.64
0	0	1	0	1	0	0	0	0.5	2.5		2.3
0	0	1	0	0	1	0	0	0	0	1	1.65
0	0	1	0	1	0	0	0	0.3	2.7		1.59
0	0	1	0	1	0	0	0	0.15	2.85		1.6
0	0	1	0	1	0	0	0	0.45	2.55		1.64
0	0	1	0	1	0	0	0	0.9	2.1		1.604
0	0	1	0	1	0	0	0	0	1		1.662
0	0	1	0	1	0	0	0	0.6	2.4		1.565
0	0	1	0	1	0	0	0	0	1		1.579
0	0	1	0	1	0	0	0	0.09	2.91		1.597
0	0	1	0	1	0	0	0	0.2	2.8		1.725

0	0	1		0	1	0	0	0	1	2		2.05
0	0	1		0	1	0	0	0	2	1		1.28
0	0	1		0	0.85	0.15	0	0	0	1		1.77
0	0	1		0	0	1	0	0	2	1		1.7
0	0	1		0	1	0	0	0	0.9	2.1		1.67
0	0	1		0	1	0	0	0	0.6	2.4		1.71
0	0	1		0	1	0	0	0	1	2		1.94
0	0	1		0	1	0	0	0	2	1		2.22
0	0	1		0	1	0	0	0	1	0		1.6
0	0	1		0	0.95	0	0	0	0.1	2.9		1.575
0	0	1		0	1	0	0	0	0.1	2.9		1.595
0	0	1		0	1	0	0	0	0.3	2.7		1.66
0	0	1		0	1	0	0	0	0.5	2.5		1.569
0	0	1		0	1	0	0	0	0	1		1.62
0	0	1		0	1	0	0	0	0.5	2.5		1.672
0	0	1		0	1	0	0	0		1		1.56
0	0	1		0	1	0	0	0		1		2.28
0	0	1		0	1	0	0	0	1	0		1.77
0	0	1		0	1	0	0	0		1		2.296
0	0	1		0	1	0	0	0		1		1.4
0	0	1		0	0	1	0	0		1		1.623
0	0	1		0	1	0	0	0		1		1.68
0	0	1		0	1	0	0	0	0.45	2.55		1.71
0	0	1		0	1	0	0	0	0.6	2.4		1.73
0	0	1		0	1	0	0	0	0.75	2.25		1.59
0	0	1		0	1	0	0	0	0.1	2.9		1.62
0	0	1		0	1	0	0	0	0.2	2.8		1.636
0	0	1		0	1	0	0	0		1		1.775
0	0	1		0	1	0	0	0	1	2		1.68
0	0	1		0	1	0	0	0	0.6	2.4		1.55
0	0	1		0	0.75	0.25	0	0	0	1		1.548
0	0	1		0	1	0	0	0		1		1.55
0	0	1		0	1	0	0	0	0	1	0	1.56
0	0	1		0	1	0	0	0	0	1	0	1.5
0	0	1		0	1	0	0	0	0	1	0	2.1
0	0	1		0	1	0	0	0	1	0	0	1.6
0	0	1		0	1	0	0	0	1	2		1.53
0	0	1		0	1	0	0	0	0	1	0	1.5
0	0	1		0	1	0	0	0	0.3	2.7	0	1.607
0	0	1		0	1	0	0	0	0	1	0	1.99
0	0	1		0	1	0	0	0	2	1	0	2.9
0	0	1		0	1	0	0	0	0	1	0	1.58
1	0		0	0	1	0	0	0	1	0	0	1.6
1	0		0	0	1	0	0	0	0	1	0	1.6
0	0		1	0	1	0	0	0	1	0	0	1.6
0	0	1		1	1	0	0	0	0	1	0	1.53
